# Flake production: A universal by-product of primate stone percussion

**DOI:** 10.1073/pnas.2420067122

**Published:** 2025-02-11

**Authors:** Tomos Proffitt, Paula de Sousa Medeiros, Waldney Pereira Martins, Lydia. V. Luncz

**Affiliations:** ^a^Technological Primates Research Group, Max Planck Institute for Evolutionary Anthropology, Leipzig 04103, Germany; ^b^Interdisciplinary Center for Archaeology and the Evolution of Human Behaviour, Universidade do Algarve, Faro 8005-139, Portugal; ^c^Programa de Pós-Graduação em Biodiversidade e Uso dos Recursos Naturais, Universidade Estadual de Montes Claros, Montes Claros, MG 39401-089, Brazil; ^d^Departamento de Biologia Geral, Centro de Ciências Biológicas e da Saúde, Universidade Estadual de Montes Claros, Montes Claros, MG 39401-089, Brazil

**Keywords:** oldowan, primate archaeology, capuchin, *Sapajus xanthosternos*, technology

## Abstract

The evolution of stone tool technology marks a significant milestone in hominin development, enabling early humans to manipulate their environments. The oldest known evidence, dating to 3.3 Ma, indicates a combination of percussive and flake production activities. Studying the archaeological signature of percussive stone tool use in living primate provides a potential analog to the origin of stone flake technology in the hominin lineage. Here, we present a yellow-breasted capuchin (*Sapajus xanthosternos*) stone tool assemblage from Fazenda Matos, Brazil, to explore the variability of the material signatures associated with percussive tool use. Our analysis of this record demonstrates many archaeological features previously associated with intentional flake production. This includes hammerstones with substantial percussive damage and a range of flaked and detached pieces. Comparative analyses with other flaked primate and hominin assemblages reveals that, unintentional flake production is a universal component of stone hammer and anvil percussive behaviors, suggesting that similar behaviors by early hominins may have led to stone flake technology and that this record may have been highly variable. To fully understand the origins of hominin stone technology, a broad spectrum of material records including both hominin and primate must be considered.

## Significance

An important avenue for understanding the origins of early hominin technology is the stone tool record of contemporary primate populations. Our research focuses on the stone tool record of yellow breasted capuchins (*Sapajus xanthosternos*) from Fazenda Matos in Brazil. We show that this species, through habitual nut-cracking activities produces a diverse fragmented lithic record, including the unintentional production of sharp-edged flakes, like those commonly associated with early hominin technology. By comparing this record across primate species, we show that flake production is a constant. This evidence highlights the potential importance of subsistence percussive behaviors as one of the possible mechanisms behind the emergence of hominin stone tool technology.

The development of stone tool technology represents a pivotal moment in human evolution, enabling hominins to modify their environments beyond their physical capabilities. Understanding the origins of this technology is fundamental to unraveling the evolution of human behavior and culture.

The earliest direct evidence of hominin technology, dated to 3.3 Ma, is the Lomekwian from in West Turkana (Kenya) (1, 2) [but see (3, 4)]. This technology features large cores and flakes retaining evidence of both percussive and flaking activities (2). Additionally, cut-marked bones from Dikikka, Ethiopia, dated to 3.34 Ma, suggest that sharp-edged flakes were used for butchery activities during this time (5). This early material record is sparse and contentious (3, 4, 6). Only with the Oldowan technocomplex [dated 2.9 to 1.6 Ma (7, 8)] tool use becomes widely abundant across the landscape and features smaller flakes produced from small cores. While the Lomekwian is associated with *Kenyanthropus platyops,* it also temporally overlaps with *Australopithecus afarensis* (1, 2). Early Oldowan assemblages (>2 Ma) are associated with both *Paranthropus* and *Homo habilis* (7, 9–11). Stone tool technology, therefore, was likely an adaptive strategy across multiple hominin species (1, 12, 13) and may have initially emerged multiple times during the Plio-Pleistocene before eventually being widely adopted during the Oldowan (10). These technologies are followed by the Acheulean [dated 1.7 to 0.3 Ma (14–16)], a technocomplex characterized by large cutting tools, large flake production and a notable increase in knapping skill (17) typically associated with *Homo ergaster/erectus*.

The emergence of stone flake production may have developed from a culture of percussion involving stone tools, similar to behaviors seen in extant primates (18–22). Pliocene and Miocene hominins likely possessed the ability to use such tools (23, 24), leading some to suggest that tool use in hominins may extend to the last common ancestor of chimpanzees and hominins, approximately 6 to 8 Ma (18, 19, 25).

The mechanisms underlying the transition from percussive behaviors to intentional flake production remain poorly understood. The accidental production of sharp-edged flakes during percussive activities, providing a visible causal relationship between flake production and hammerstone use is, however, a prevailing hypothesis (20, 22, 26, 27). Studies of modern primates show that hominin stone flake production may have emerged accidentally as a by-product of such percussive activities (22, 28–31).

Chimpanzees (*Pan troglodytes verus),* capuchins (*Sapajus libidinosus, Cebus capucinus*), and long-tailed macaques (*Macaca fascicularis)*, use stone tools for various tasks (32–36), leaving durable material signatures (30, 37–40). Both, bearded capuchins from Serra da Capivara National Park (SCNP), Brazil, and long-tailed macaques (Phang Nga National Park, Thailand) unintentionally produce large quantities of sharp-edged flakes similar to those found in Plio-Pleistocene hominin archaeological assemblages (28, 29) through stone on stone percussion (28, 41) and nut cracking (22, 29).

**Fig. 1.**
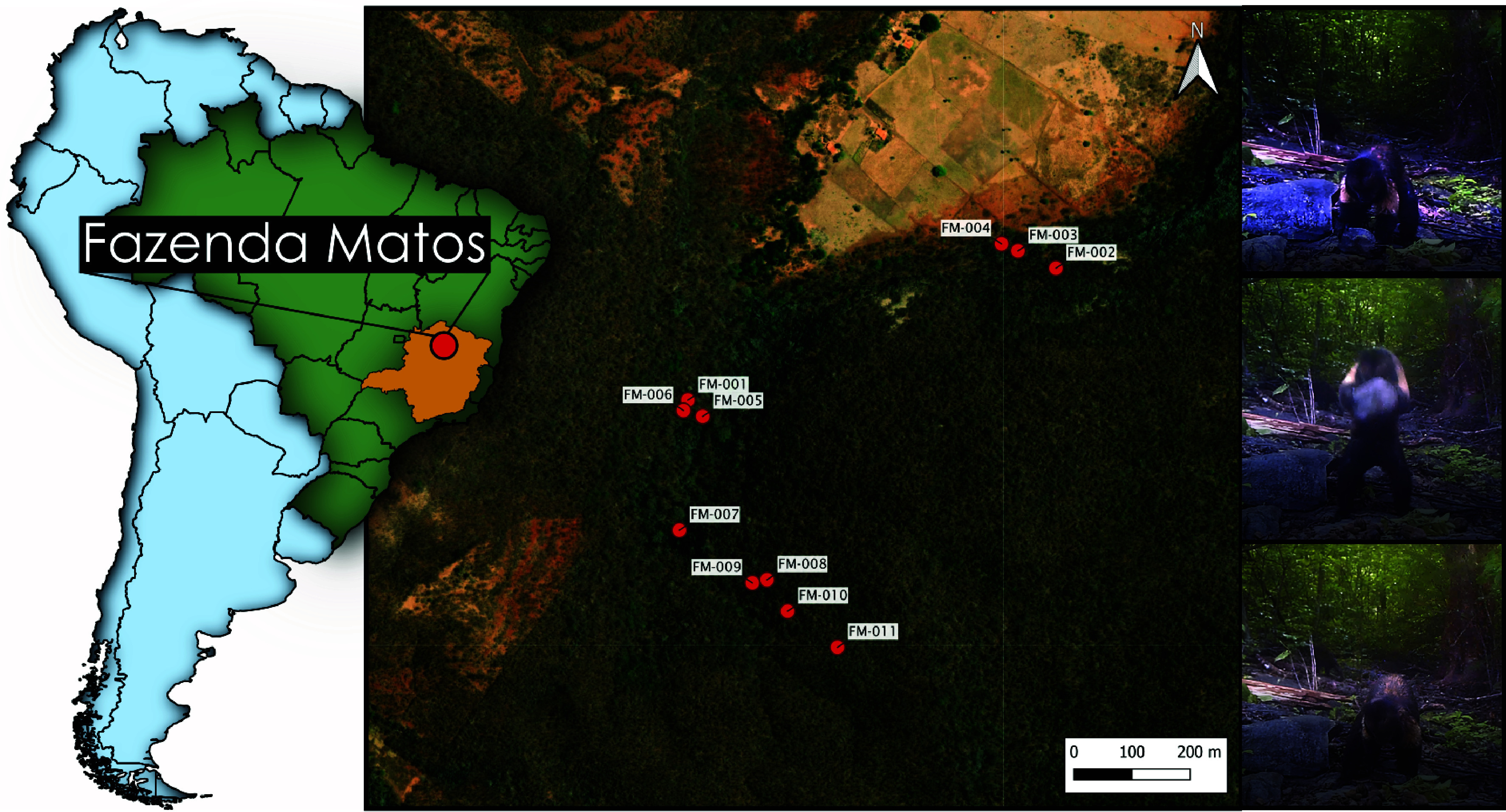
Location of Fazenda Matos (red), Brazil (green) in the state of Minas Gerais (yellow), and the location of all nut-cracking sites included in this study alongside images of yellow-breasted capuchin stone tool use captured by camera trap within the study area.

Anecdotal reports indicated that yellow-breasted capuchins (*Sapajus xanthosternos*) in Brazil also use stone tools for nut cracking (42). Here, we report on the first nut-cracking stone tool assemblage of a wild population of yellow-breasted capuchins at Fazenda Matos, Brazil (Fig. 1). We directly compare the assemblage with the material signature of long-tailed macaques in Thailand. The similarity of raw material allows us to explore whether the same behavior undertaken by species occupying different environments and separated by millions of years of evolutionary divergence produces a similar material record. Combined with evidence of other modern primate flaked lithic assemblages, it is now clear that unintentional sharp edged flake production is a universal signature of percussive stone tool use. Finally, we compare all primate flaked assemblages to known Plio-Pleistocene hominin assemblages and suggest that while the hypothesized material signature associated with the emergence of stone flake technology would be identifiable, in terms of technological attributes, it may have exhibited considerable variability compared to the Oldowan.

## Results

### Behavior.

Yellow-breasted capuchins at Fazenda Matos crack nuts in an upright bipedal position, holding a hammerstone in both hands. To strike, they extend to full height, raising the stone to head level, then forcefully bring it down onto the nut in an uncontrolled motion. Occasionally, they jump slightly during the upward movement, possibly to increase momentum (Movie S1).

### Yellow-Breasted Capuchin Nut-Cracking Lithic Assemblage.

**Fig. 2.**
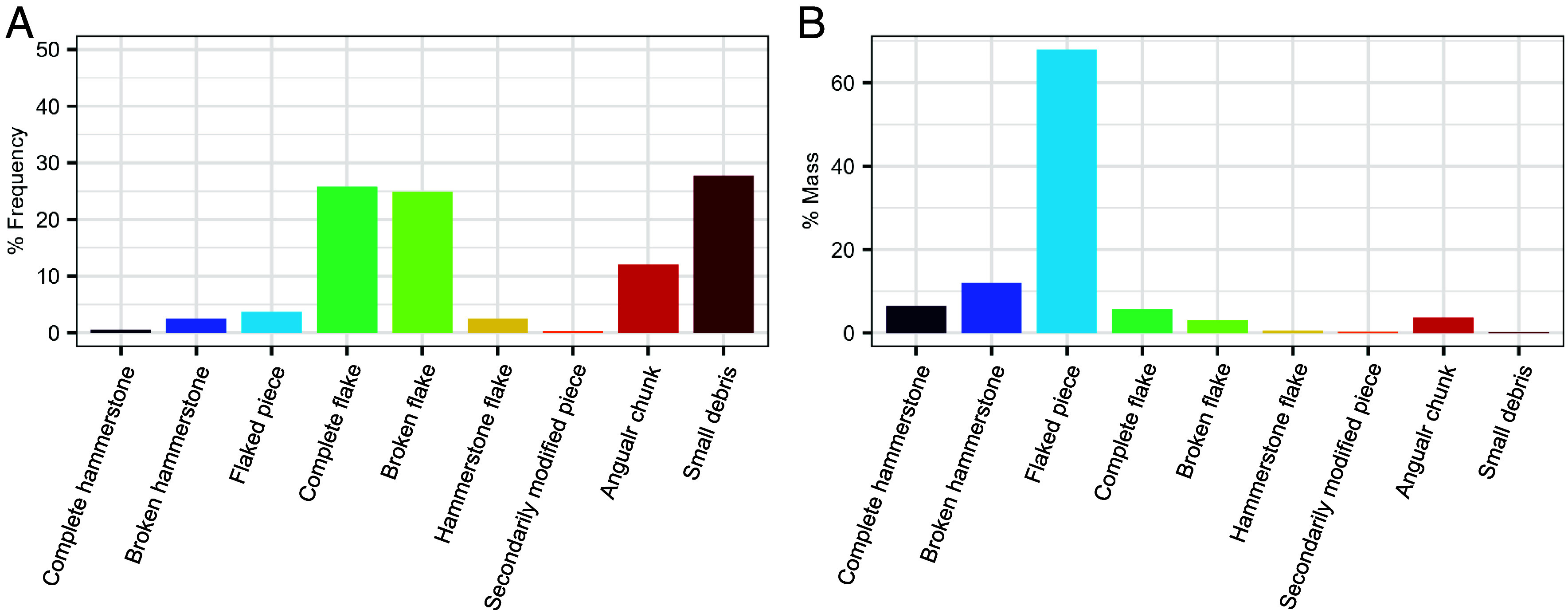
Relative frequencies (*A*) and mass (*B*) of all technological categories represented in the nut-cracking lithic assemblage from Fazenda Matos.

Capuchin nut cracking on stone anvils produces a highly fragmented lithic assemblage (n = 357) due to the isotropic nature and tabular morphology of the raw material. The assemblage includes detached pieces (93.27%, n = 333) (complete and broken flakes, hammerstone flakes, small debris, and angular chunks) and percussive tools (6.72%, n = 24) (hammerstones, broken hammerstones, and flaked hammerstones) (Fig. 2). The raw material of the assemblage consists of a fine-grained siltstone with a mean (Schmidt hammer) hardness of 44.8 (range: 27 to 91.5, SD: 7.6) (*SI Appendix,* Fig. S1). All technological categories are represented across the total assemblage, though not at each individual nut-cracking site (*SI Appendix*, Table S1).

### Hammerstones and Flaked Pieces.

While percussive tools comprise a small portion of the assemblage by count (n = 24, 6.72%), they contribute substantially to its total mass (86.49%, 21.76 kg) (Fig. 2).

**Fig. 3.**
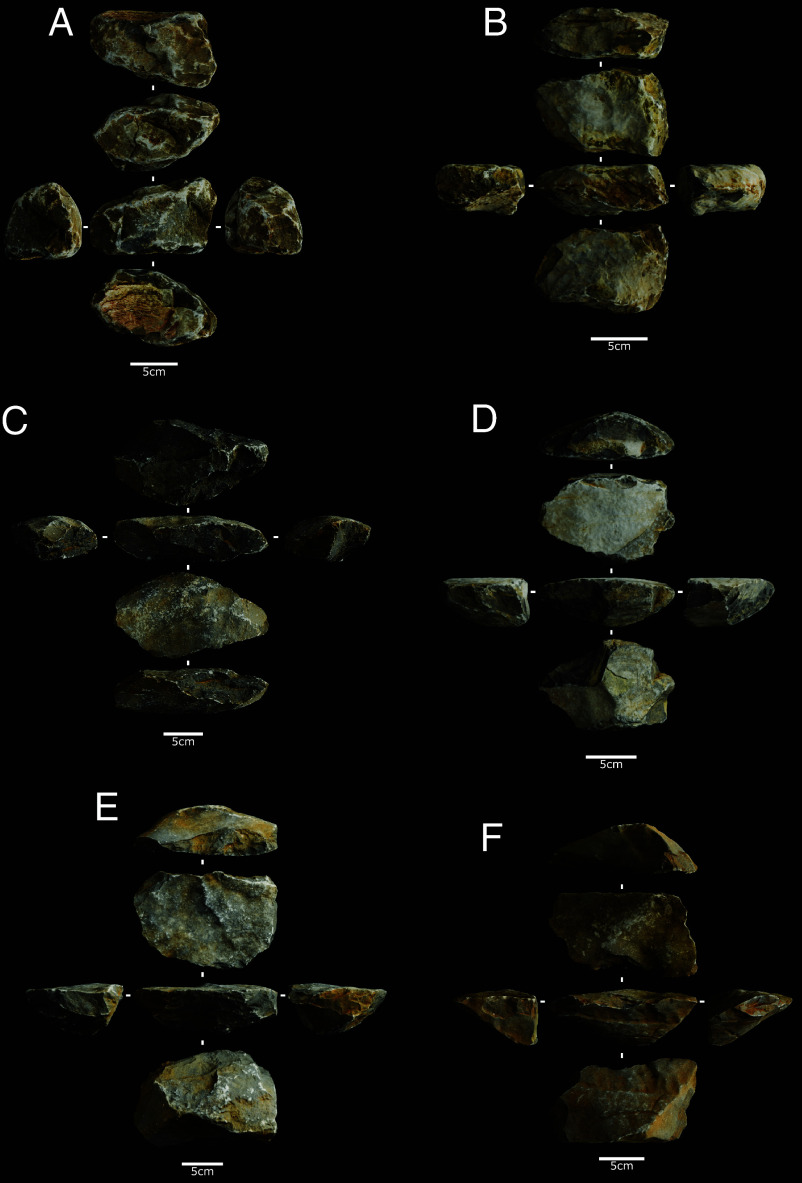
Examples of fine-grained siltstone complete hammerstones (*A* and *B*) and flaked pieces (*C*–*F*) from used for nut cracking by yellow breasted capuchins at Fazenda Matos.

There is no difference in the size, weight, or morphology of complete hammerstones and flaked pieces compared to the locally available raw material (*SI Appendix,* Fig. S2 and Table S2). The assemblage contains two complete hammerstones, averaging 130.6 × 73.4 × 53.2 mm and 815.8 g, exhibiting similar percussive damage across two to six active planes on ridges, flat, and convex surfaces, characterized by superimposed impact points and varying levels of crushing including along their edges (Fig. 3 *A* and *B*).

Nine broken or fragmented hammerstones, averaging 97.9 × 68.2 × 41.5 mm and 335.8 g, display clustered (44.4%), dispersed (44.4%), or isolated (11.1%) percussive damage with impact points (100%) and crushing (66.7%) on ridges (77.7%) and flat surfaces (66.6%), with most exhibiting a single use–related break (55.5%) and some showing multiple fractures (44.4%).

Flaked pieces, though few in number, comprise 68% of the assemblage’s total mass, averaging 148.8 × 94.8 × 20.9 mm and 254 g (up to 780.3 g), and while similar to complete hammerstones in length, thickness, and mass, they are significantly wider.

All flaked pieces exhibit tabular or plano-convex morphologies with natural 90° or acute angles between planes and show extensive percussive damage across multiple surfaces (predominantly on two flat opposed planes), indicating their use as frequently rotated hammerstones with a preference for larger flat surfaces for percussion (Fig. 3 *C*–*F*).

The active surfaces and natural acute edge angles facilitate superimposed recurrent flake detachments on adjoining surfaces, with an average of 9.15 flake scars per piece (119 total scars >10 mm), typically wide and short (mean 27.9 × 39.6 mm, elongation 0.75) but sometimes elongated or large (>50 mm), indicating that nut cracking can result in substantial detachments (*SI Appendix,* Fig. S3).

The flaked pieces exhibit varied percussive damage and edge battering across multiple active surfaces, with flake detachments occurring where these surfaces intersect adjoining planes at acute angles, often resulting in contiguous, superimposed, and invasive unidirectional flake scars along the flaking surface (Fig. 3 *C*–*F*). This is clearly evidenced through a number of refit sets (*SI Appendix*, *Supplementary Information 1* and Fig. S4 and Movies S2–S4).

Various flake removal patterns are present. These are predominantly unifacial and unidirectional (77%), with both abrupt (46.2%) and acute (30.8%) edge angles, while bifacial (15.4%) and multifacial (7.69%) patterns are also present, reflecting diverse hammerstone use and rotation during nut-cracking activities (*SI Appendix*, Tables S4 and S5).

### Complete Flakes.

The complete flakes measure on average 39.7 × 25 × 8.9 mm and weigh 15.8 g and are typically wide and short (elongation ratio 0.81), though larger and more elongated flakes are also present in the collection (*SI Appendix,* Fig. S5 and Table S3).

Detached flakes exhibit a mean exterior platform angle of 89.04° and diverse bulb morphologies (57.6% diffused, 15.2% marked, 25% absent), suggesting a mix of conchoidal and wedge-initiated detachments in the assemblage.

Flake platforms average 24.1 × 7.2 mm, predominantly central (59.8%), cortical, and rectilinear (81.5%), with some noncortical or partially cortical platforms indicating extended reduction sequences, while platform characteristics [mostly flat, some unifaceted, rarely bi- or multifaceted (*SI Appendix*, Table S6)] reflect the simple, ad hoc nature of the detachment process.

Most flakes exhibit feather terminations (67.4%), while hinge, plunging, and step terminations are also present (*SI Appendix*, Table S6). These terminations suggest varying impact forces during flaking and the lack of surface management. Step scars appear on the dorsal surface of multiple flakes but are not universally observed (34.8%), with single instances more common than stacked scars (*SI Appendix*, Table S6).

**Fig. 4.**
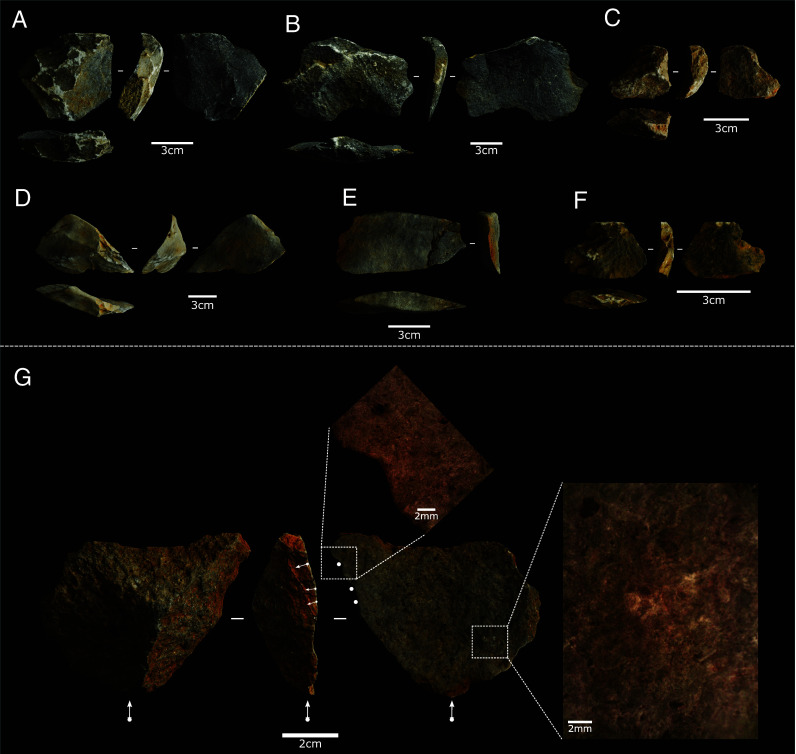
Examples of complete flakes (*A*–*F*) and the single retouched flake (*G*) produced unintentionally through nut cracking at Fazenda Matos.

Most flakes in the assemblage possess either cortical or mostly cortical (>50%) dorsal surfaces. However, most flakes fall into the early stages of reduction (88.1%; Toth flake categories I to III). Percussive damage is rare on dorsal surfaces of flakes (23.9%), and multiple scars suggest recurring detachment patterns, primarily unidirectional (88.1%) with some opposed and transversal scar directionality (Fig. 4 *A*–*F* and *SI Appendix,* Fig. S6 and Table S6).

### Secondarily Modified Pieces.

A large cortical flake displays clear percussive damage on both its dorsal and ventral surfaces, indicating its use as a percussive tool after detachment. The damage consists of a small cluster of impact points on the raised ventral area. Additionally, three contiguous noninvasive removals from its distal right edge show distinct impact points and feather terminations (Fig. 4*G*). Although its production was not observed, its location at a nut-cracking site, away from waterways and channels, along with no visible taphonomic disturbances such as trampling, suggests that capuchin monkeys reused it as a small percussive tool.

## Comparison of Yellow Breasted Capuchin and Long-Tailed Macaque Lithic Assemblages

### Behavior.

When comparing nut-cracking techniques, yellow-breasted capuchins tend to be less controlled compared to long-tailed macaques. Macaques typically sit down and use one hand to lift the hammerstone from below to mid-waist before flipping it over and striking in a controlled motion, using the free hand to shield the nut. This method and posture likely enhances the precision of the nut-cracking process. While capuchins employ a dynamic, two-handed forceful technique, with full body extension.

### Material Signature Comparison Across Species.

**Fig. 5.**
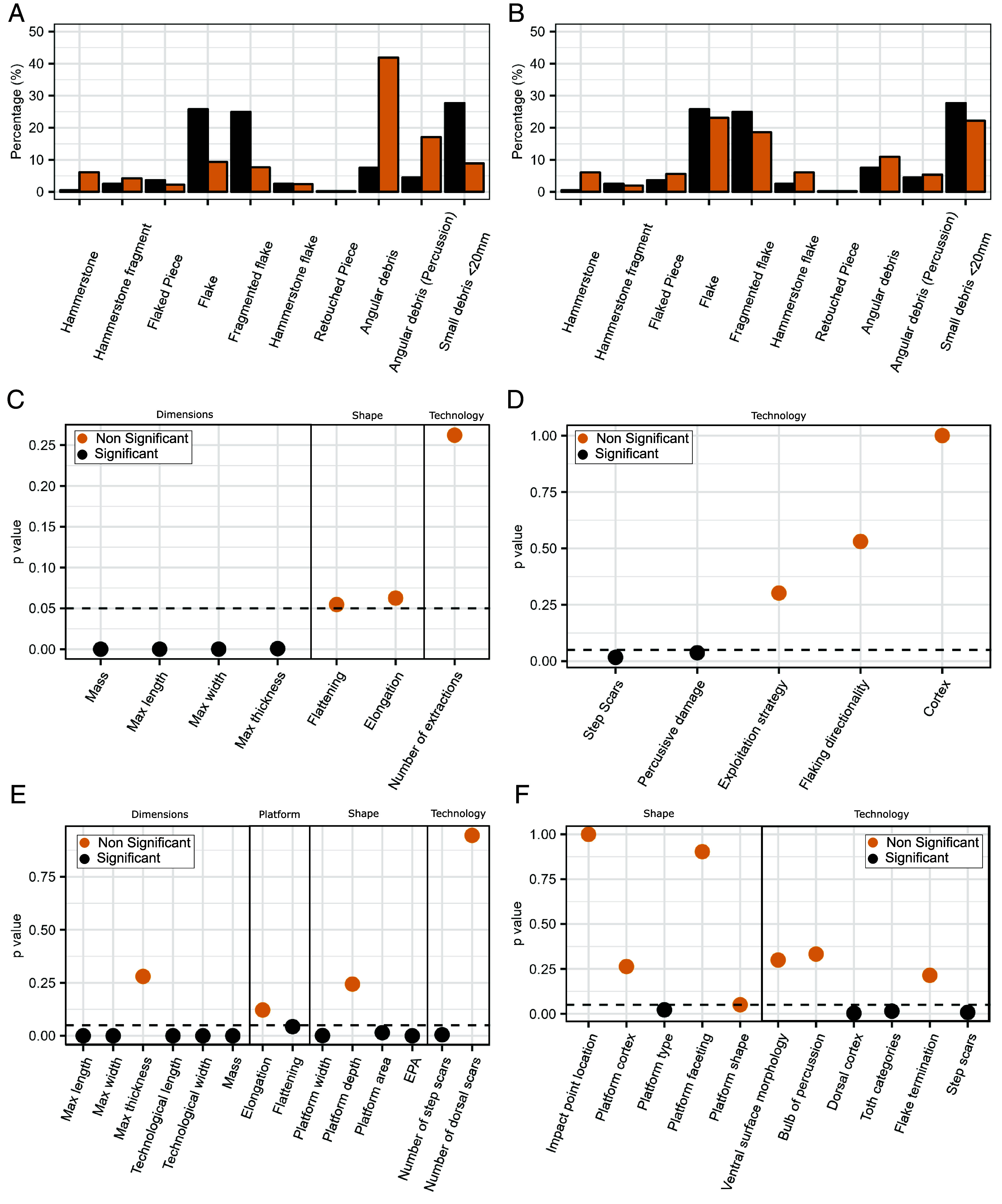
Comparative analysis of the lithic assemblages from Fazenda Matos and Lobi Bay. (*A*) Comparison of the relative frequencies of all technological categories between Fazenda Matos and Lobi bay separated by all raw materials and (*B*) only comparing the high quality raw material at Lobi Bay. Comparison of flaked pieces (*C* and *D*) between Fazenda Matos and Lobi Bay showing significant and nonsignificant *P*-vales for the results of Mann–Whitney *U* tests on (*C*) quantitative attributes and (*D*) Chi-Square tests for attribute data. Comparison of flake data (*E* and *F*) between Fazenda Matos and Lobi Bay showing significant and nonsignificant *P*-vales for the results of Mann–Whitney *U* tests on quantitative data (*E*) and Chi-Square tests for attribute data (*F*). (*P* = 0.05 is represented by a horizontal dashed line).

Significant differences in technological categories between the capuchin assemblage of Fazenda Matos and the long-tailed macaque assemblage from Lobi Bay are clear, with Fazenda Matos showing fewer angular pieces and hammerstones, but more complete and fragmented flakes, as confirmed by a chi-square test (χ^2^ = 34.701, df = 9, *P* < 0.001). However, the Lobi Bay assemblage consists of two different weathering stages of limestone material. When focusing only on highly isotropic raw material, the assemblage comparison changes, showing a significant difference mainly due to Lobi Bay’s abundance of complete hammerstones, unlike Fazenda Matos (χ^2^ = 238.67, df = 9, *P* < 0.001), yet both assemblages are remarkably similar in the relative frequency of other technological categories (Fig. 5 *A* and *B* and *SI Appendix*, Table S7).

Flaked pieces from Fazenda Matos (capuchin) are significantly larger and heavier than those from Lobi Bay (macaque), with no notable difference in elongation or flattening between the two collections (*SI Appendix,* Fig. S7). The Fazenda Matos collection, despite a higher occurrence of step scars, shares similar technological characteristics with the Lobi Bay collection, including cortical coverage and flake removal patterns, with predominantly unifacial exploitation patterns across both assemblages (Fig. 5 *C* and *D* and *SI Appendix*, Tables S8–S10).

Complete flakes from Fazenda Matos are notably larger in length, width, and weight, with wider platforms and higher EPA values, although platform depth is consistent with those from Lobi Bay (*SI Appendix*, Table S11).

Out of 14 identified technological attributes on complete flakes, significant differences in only four aspects—dorsal cortex coverage, reduction stages, platform type, and step scar quantity—distinguish the two assemblages (*SI Appendix*, Table S12).

The flakes from Fazenda Matos and Lobi Bay show similarly low levels of reduction. However, Fazenda Matos has a higher frequency of Stage III flakes, while Lobi Bay contains more Stage II and Stage V flakes.

The Fazenda Matos flake assemblage features more lineal platforms, while Lobi Bay has more punctiform ones, yet both predominantly have clear, cortical platforms with central impact points and similar platform shapes and faceting.

Flakes from Fazenda Matos possess significantly more step scars on their dorsal surfaces (Fig. 5*E* and *SI Appendix*, Tables S12 and S13) compared to Lobi Bay, mirroring the increased frequency of step scars on flaked pieces.

Both collections consist mainly of flakes with cortical, flat platforms and a predominantly clean flake terminations, though Fazenda Matos has more hinge-terminated flakes, with both showing diffuse percussion bulbs and flat or concave undersides (Fig. 5 *E* and *F*).

### Comparison with the Archaeological Record.

**Fig. 6.**
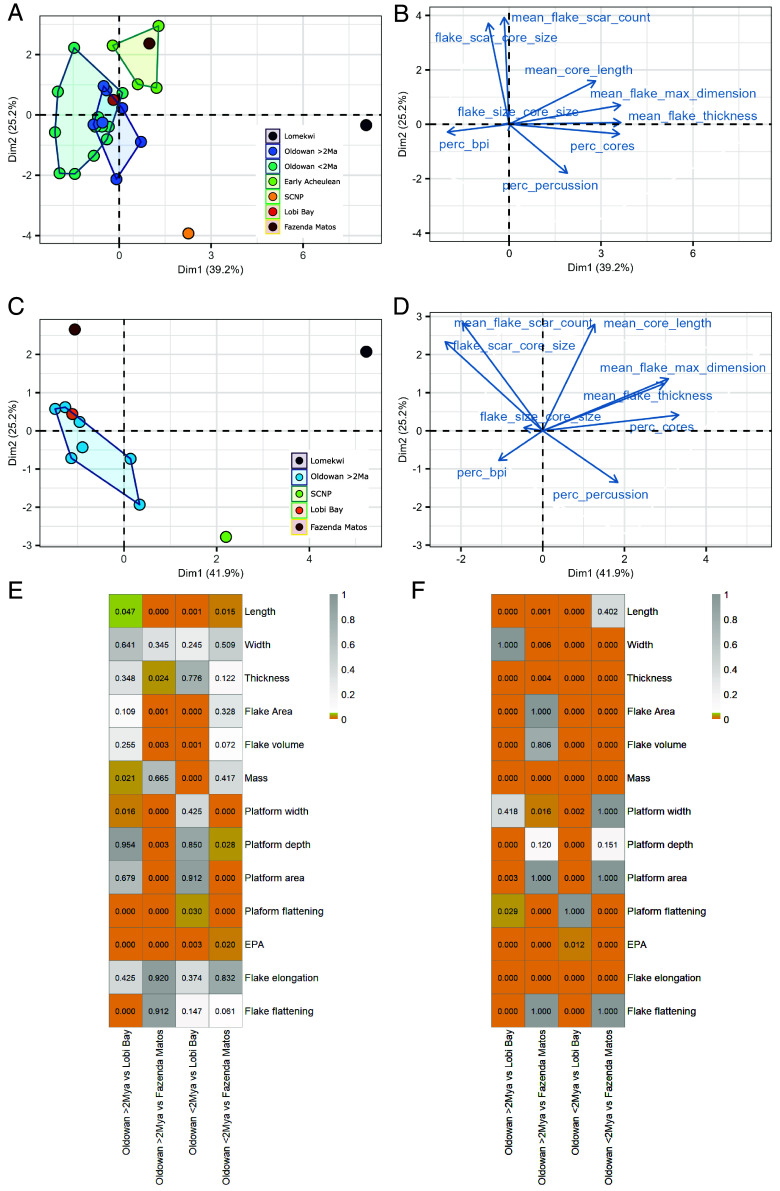
Comparative analysis of primate flaked assemblages with Plio-Pleistocene artifacts using a principal component analysis (PCA) based on technological aspects of primate and Plio-Pleistocene archaeological assemblages (*SI Appendix*, Table S14) and pairwise Brown Forsythe and Dunn’s tests. (*A*) Plot of principal component (PC) 1 and PC2 of flaked primate assemblages alongside Lomekwi, Oldowan, and Early Acheulean assemblages with associated loadings (*B*) for PC1 and PC2. (*C*) Plot of PC1 and PC2 for all flaked primate assemblages alongside Lomekwi and Oldowan assemblages older than 2 Ma with associated loadings (*D*) for PC1 and PC2. (*E*) Results of a pairwise Brown–Forsythe test showing significant differences in assemblage variation for all quantitative attributes of flakes between Fazenda Matos, Lobi Bay, and Oldowan flakes. (*F*) Results of a pairwise Dunn’s test indicating significant differences in values for all quantitative attributes of flakes between Fazenda Matos, Lobi Bay, and Oldowan flakes. Significant *P*-values, ranging from <0.001 to 0.05, are represented by a gradient from orange to yellow, while nonsignificant values (>0.05) are shown from white to gray.

A PCA shows that the technological traits of the Fazenda Matos and Lobi Bay assemblages more closely cluster with Oldowan and Early Acheulean assemblages compared to either the stone-on-stone assemblage from SCNP or the Lomekwian assemblage. While the Lobi Bay assemblage falls within the range of variation for the Oldowan, the assemblage from Fazenda Matos falls within the range of Early Acheulean assemblages (Fig. 6*A*). These are differentiated by the flake scar-to-core size ratio, mean flake scar count, and core size (Fig. 6*B*).

When focusing only on Oldowan samples over 2 Ma old, the Lomekwian site, and primate assemblages, the PCA plot shows significant variation with each assemblage occupying distinct areas (Fig. 6 *C* and *D* and *SI Appendix*, Table S14).

While, Fazenda Matos flakes are significantly longer compared to Oldowan flakes greater than 2 Ma, Lobi Bay flakes are significantly shorter compared to all Oldowan flake assemblages, a pairwise Brown Forsythe test reveals that primate flake assemblages also exhibit greater diversity than Oldowan assemblages in flake dimensions and platform metrics, with notable variability in flake length, area, volume, mass, and platform dimensions and flattening (*SI Appendix*, Tables S15 and S16 and Fig S8).

Furthermore, primate flake assemblages both possess significantly more variable EPA values as well as being significantly larger (χ^2^ = 159.63, df = 3, *P* < 0.001) compared to Oldowan flakes older and younger than 2 Ma. Specifically, Fazenda Matos flakes have higher EPA values than those from Lobi Bay, and both exceed those in Oldowan assemblages (*SI Appendix,* Fig. S11). Technological attributes of flakes show considerable variation between primate and Oldowan flakes. A chi-square test reveals significant differences between the two groups in the number of platform facets, dorsal scars, directions of dorsal scars, and the degree of dorsal cortex (*SI Appendix*, Table S17). Adjusted residuals indicate that primate flakes are significantly more likely to lack platform faceting compared to Oldowan flakes, both older and younger than 2 Ma (*SI Appendix*, Table S18 and Fig. S9*A*). Additionally, primate flakes exhibit a higher frequency of flakes with no dorsal scars (*SI Appendix*, Table S18 and Fig. S9*B*). Fazenda Matos flakes, in particular, show significantly less diverse dorsal scar patterns than Oldowan flakes older than 2 Ma (*SI Appendix*, Table S18 and Fig. S9*C*). Finally, primate flakes are significantly more likely to retain 100% dorsal cortex compared to Oldowan flakes (*SI Appendix*, Table S18 and Fig. S9*D*).

Significant differences in exploitation patterns and the number of flaked faces distinguish primate and Oldowan flaked pieces. Primate flaked pieces predominantly exhibit unifacial reduction, while Oldowan assemblages show more diverse bifacial and multifacial strategies (x^2^ = 153.63, df = 52, *P* < 0.001; *SI Appendix*, Fig. S10 and Tables S19 and S20).

## Discussion

Exploring the variation in percussive stone tool evidence among primates and hominins is crucial for understanding the evolution of technology within our lineage. The earliest known archaeological evidence indicates a combination of flake production and percussive activities (1, 2). The persistence of percussive technology throughout the Plio-Pleistocene hominin record (43–48), coupled with its prevalence among stone tool-using primates (32–34, 49–52), suggests a broader spectrum of percussive behaviors from which stone flake technology may have emerged (53, 54).

Previously, four species of wild primates have been known to use stone tools. Only bearded capuchins and long-tailed macaques have been described to repeatedly produce flaked pieces and sharp-edged flakes (22, 28, 29). This is solely dependent on the raw material used for the percussive task performed (22, 29, 55). While yellow-breasted capuchins have previously been reported to use stone tools (42), our study reveals a similarly diverse material signature associated with this nut-cracking behavior. This signature, like those of other primate assemblages produced on homogenous and isotopic raw materials, includes hammerstones that show extensive percussive damage but minimal alteration of their active surface morphology. Additionally, the assemblage contains various fragmented pieces, including flaked and detached items, with sharp-edged flakes being especially notable. These artifacts share a wide range of dimensional, shape, and technological attributes with Plio-Pleistocene lithic assemblage.

Moreover, despite being a singular instance, the discovery of a detached piece with secondary flake removals, at Fazenda Matos indicates that in certain scenarios, owing to their larger dimensions, flakes will be repurposed as hammerstones. This may lead to the inadvertent creation of an artifact type (retouched tool) consistently linked with deliberate tool production in the hominin archaeological record. This artifact’s presence within the Fazenda Matos assemblage marks the only reported occurrence of a monkey species producing and using a stone tool, beyond merely selecting an unaltered stone for use. Previously, such behavior had solely been observed in chimpanzees (31) and hominins (9).

Combined with recent reports of flake production from percussive activities by bearded capuchins and long-tailed macaques, it is evident that the unintentional production of flakes and flaked pieces is a shared component of the material signature of percussive behaviors in wild primates. While environmental factors such as raw material homogeneity and availability may introduce variability, the ubiquity of this material signature underscores its significance.

The yellow-breasted capuchin assemblage from Fazenda Matos expands our sample of fragmented lithic material beyond bearded capuchins and long-tailed macaques and suggests that certain lithic artifacts should not automatically be interpreted as intentional artifacts in the archaeological record. Specifically, this includes large, flaked pieces with impact points, contiguous, superimposed, often invasive flake detachments, possessing percussive damage on one or more planes, and large flakes with clear cortical and noncortical platforms, lacking platform battering, with multiple dorsal scars, and external platform angles of 90° or less. Furthermore, our results suggest that the size of artifacts, such as cores and flakes, does not necessarily correlate with the tool user’s body size and morphology or advanced technical skills. The dimensions of these artifacts seem to be more closely associated with both the original dimensions of the raw material and the raw materials underlying fracture mechanics.

It has been theorized that tool use may have extended beyond the known hominin archaeological timeline into the early and middle Pliocene, potentially reaching back into the Miocene era (18, 56). The results of this study suggest that regardless of fossil hominin or living primate species, if percussive technology was part of their adaptive toolkit, their ecological niche included encased food sources and their available raw material was homogenous and isotropic (22, 30), tool users likely left behind a corresponding and identifiable material record.

The emergence of stone flake technology in the hominin lineage might be linked with multiple instances of convergent evolution (10), with the earliest archaeological assemblages attributed to at least four different hominin species (2, 5, 10, 57–59). by comparing the material signatures of yellow-breasted capuchins at Fazenda Matos and long-tailed macaques at Lobi Bay in Phang Nga National Park, we demonstrate the material record of similar behaviors performed by two species separated by significant environmental differences and millions of years of evolution share similarities in both assemblage composition as well as technological similarities. At Fazenda Matos detached pieces, however, are significantly larger than artifacts from Lobi Bay. This may be associated with a greater degree of force employed by yellow-breasted capuchins during their bimanual manipulation of their tools, affecting the observed reduction levels between the two assemblages. The repeated removal of larger flakes early in the detachment sequence at Fazenda Matos leads to higher levels of reduction. However, the increased prevalence of thin flake platforms and step scars at Fazenda Matos attest to a lack of control over the larger hammerstones at the point of impact, making it more likely to impact close to the edge of the hammer or anvil with inconsistent force transmission.

While yellow-breasted and bearded capuchins, as well as long-tailed macaques, produce extensive lithic assemblages similar to those typically associated with Plio-Pleistocene hominin lithic assemblages in terms of artifact categories, these assemblages do not form a coherent cluster when compared using various technological attributes to both Oldowan and Early Acheulean assemblages. Only the long-tailed macaque assemblage overlaps with Oldowan assemblages older than 2 Ma. The Fazenda Matos assemblage is distinguished by attributes associated with core size and number of flake scars, placing it within the range of Early Acheulean assemblages albeit clearly not associated with such a technocomplex. The SCNP stone-on-stone assemblage is characterized by a relatively greater frequency of percussive artifacts and sits apart from both the Fazenda Matos and the archaeological assemblages of hominins.

This variation is also reflected specifically in the flake assemblages. Both flake assemblages produced by yellow-breasted capuchins and long-tailed macaques are more diverse across almost all dimensions compared to Oldowan assemblages older and younger than 2 Ma. While individual flakes from these assemblages have been demonstrated to fall within the variation range of Oldowan technology (29), our results suggest that at the assemblage level, these records differ. Primate flake assemblages generally possess larger external platform angles compared to Oldowan assemblages, with a reduced variation in scar patterning and platform faceting, with these being largely unidirectional and nonfaceted, reflective of the simple exploitation patterns observed on the capuchin flaked pieces, similar to the earliest archaeological record (2).

At a wider assemblage level, this signature is also characterized by the presence of flaked pieces with evidence of percussion on one or more surfaces, however, also exhibiting unidirectional superimposed, occasionally invasive, scar patterns with frequent step terminations. Although modern primate percussive technology possesses less elaborate exploitation strategies compared to many Oldowan assemblages—where flakes are repeatedly detached using bifacial, polyhedral, and multifacial methods (*SI Appendix*, Fig. S10 and Tables S19 and S20) (60, 61)—its study offers insights into identifying material records not resulting from intentional flake production. While bearing the technological hallmarks of intentionality, these unintentionally flaked pieces suggest that with an increase in percussive evidence in an assemblage, there is higher likelihood that percussion, rather than knapping is the associated underlying behavior. Intentionality of flake production in such assemblages, would therefore, require additional evidence beyond such artifacts and technological attributes. In all primate assemblages, flake production is an unintentional but consistent by-product of percussive activities, when raw material isotropy is high. This contrasts with the hominin record, where post-2 Ma evidence indicates intentional selection of raw materials for specific purposes. Fine-grained, isotropic materials like chert and quartzite were often chosen for flake production and secondary retouch, while harder igneous materials were commonly used as hammerstones (60–62), reflecting a seemingly strategic approach to raw material use. Even in earlier (pre-2 Ma) Oldowan contexts, flake production dominates, with some evidence of preferential selection of igneous materials based on their intrinsic properties or morphology (63) and a lack of percussive battering on core platforms (10, 60, 63), indicating that intentional flake production was a central activity during the Oldowan.

If the by-product hypothesis stands as a valid mechanism associated with the emergence of stone flake technology (20, 22), our results suggest that it could have occurred within the behavioral repertoire of any stone tool-using hominin or primate undertaking percussive activities, leaving behind a recognizable material in the archaeological record. However, the archaeological signature of these behaviors may be diverse. Therefore, in the pursuit of understanding the emergence of stone technology and its associated material signature, one should not solely focus on Oldowan or Lomekwi-type assemblages but remain open to an archaeological record more closely resembling that of modern-day primate material.

## Materials and Methods

### Area of Study.

Yellow-breasted capuchin monkeys (*S. xanthosternos)* are classified as Critically Endangered both nationally and globally, as per the IUCN Red List (64). Previously believed to be endemic to Brazil’s Atlantic Forest, they are now known to inhabit the Caatinga and transitional biomes, particularly in dry forest regions, confined to mountainous and hilly terrains with forests (65). The species’ geographic range is delineated by the São Francisco River to the north and west, the Jequitinhonha River to the south, and the Atlantic Ocean to the east (65).

Fieldwork was conducted at Fazenda Matos (Matos’ farm), situated in the Santa Rosa de Lima district of Montes Claros, northern Minas Gerais state due to reported instances of stone tool usage by yellow breasted capuchins (42), and personal communications with local community members in the region. Fazenda Matos is located approximately 59 km north of Montes Claros city (Fig. 1). Covering about 1,000 ha, the farm comprises a forest fragment with native vegetation, interspersed with areas affected by human activity such as logging. The landscape features seasonal deciduous or dry forest (Mata Seca) with rocky outcrops, predominantly characterized by limestone massifs. There are two nut tree species used by the capuchins in this region, Guariroba (*Syagrus oleracea*) and Cansanção (*Cnidosculus pubescens*).

### Behavioral Recording.

As the capuchins at Fazenda Matos are nonhabituated to human observers, direct behavioral observations are not possible. All behavior recording was undertaken using remote camera traps (Bushnell) located at known nut-cracking sites. Cameras were placed facing naturally occurring stone outcrops that were previously used as anvils for nut cracking. Captured footage was used to describe the nut-cracking behavioral patterns.

### Field Data Collection.

Out of 125 identified nut-cracking sites, 11 sites (FM001 to FM011) within the home range of a yellow-breasted capuchin group were sampled for stone tools and associated fragmented assemblages (Fig. 1). A site was identified as a nut-cracking location based on the presence of a central stone anvil, one or more stones showing percussive damage, and fresh cracked nut shells within a 1 square meter with the anvil at its center. At each site, all forest leaf litter and organic material were removed by hand. Stone artifacts within a one-meter square area, cantered on the anvil, were then collected for analysis.

## Lithic Analysis

### Raw Material.

Raw materials were identified according to their physical characteristics. Furthermore, throughout the study area, raw material hardness was collected using a Schmidt Hammer. Hardness samples were collected from large, embedded rocks and boulders of the same raw material that make up the hammerstones, anvils and associated fragmented assemblage. Each sampled rock was subjected to 10 Schmidt hammer readings, which was then averaged to produce an overall hardness value for the stone.

### Lithic Analysis.

All collected lithics from nut-cracking sites were measured, weighed, and subjected to a techno-typological classification and analysis (*SI Appendix*, *Supplementary Information 1*), a method successfully applied to both primate and hominin flake lithic assemblages (28, 29, 66). Each lithic was classified into one of the following categories: complete hammerstone, broken hammerstone, flaked piece (in the case of primate percussive artifacts all flaked pieces are percussive objects with flake removals), complete flake, broken flake, retouched piece, angular debris, small debris, and unmodified stones following established methods by ref. 29. Percussive damage on hammerstones and flaked pieces was recorded according to established protocols for percussive technology (67–69). A range of quantitative and qualitative attributes, associated with dimensions, shape, and technology, were recorded for both complete flakes and flaked pieces. Based on platform and dorsal cortex coverage, each flake was classified into stages I to VI (70), representing their relative stage in the reduction process, with stages I to III indicating early and stages IV to VI indicating later reduction stages.

To identify whether hammerstones at Fazenda Matos were selected based on dimensions, mass, or morphology from the natural stones available, we compared the maximum dimensions and mass of all hammerstones and flaked hammerstones with naturally occurring stones collected along transects throughout the study region. Natural stones were collected and measured across 19, one meter square sampling regions within the study area. A total of 167 stones were collected using this method. Using the maximum measurements, we classified the morphology of all hammerstones and natural stones following the particle shape analysis proposed by Sneed and Folk (71) based on maximum dimensions, these were compared using a chi square test.

### Comparative Analysis.

The lithic assemblage from Fazenda Matos was subjected to a series of comparative analyses. First, to determine whether similar behaviors using isotropic raw materials by different primate species in various ecological conditions produce differing material signatures, the Fazenda Matos assemblage was directly compared to the recently documented long-tailed macaque nut-cracking assemblage from Lobi Bay in Phang Nga National Park (29).

Observations of this macaque group reveal the use of large, embedded limestone boulders as anvils for cracking oil palm nuts, employing limestone hammers (29, 36, 55). This nut-cracking behavior results in a fragmented lithic assemblage due to hammers and anvil breakages. The resultant detached and flaked pieces artifacts fall within the same range of dimensions and technological attributes to those identified in hominin Plio Pleistocene archaeological sites (29, 49). In the macaque assemblage, two distinct qualities of raw material are evident. The first is a fine-grained (high quality), homogeneous limestone, which accounts for most of the flaked and detached pieces. The second is a highly fragmented, nonhomogeneous weathered limestone (low quality), constituting most of the angular debris in the assemblage (29, 55).

Second, to explore the degree of variability between primate percussive and hominin flaked assemblages, all known flaked primate lithic assemblages (formed by percussive behaviors) were directly compared to a large sample of hominin lithic assemblages from the Plio-Pleistocene (72). For this comparison, we included primate assemblages from Fazenda Matos and Lobi Bay (29), and published lithic assemblages associated with bearded capuchin stone-on-stone percussion from SCNP in Brazil (28). Although the stone-on-stone assemblage does not stem from nut-cracking behavior, it represents a lithic assemblage dominated by flaked and detached pieces derived from a percussive activity. As such, it serves as a point of variation in the material signatures associated with percussive technology. Furthermore, we directly compare primate and Plio-Pleistocene flaked pieces in terms of exploitation directions, exploitation patterns, and number of flaked surfaces. Plio-Pleistocene flaked pieces data were taken from a previously published analysis (29).

### Statistical Analysis.

All quantitative measures were compared using a Mann–Whitney U test, followed by a post hoc Dunn’s test with a Bonferroni correction to identify sources of significant variation. Comparisons of technological attributes were undertaken using a chi-square test with subsequent analysis of the adjusted residuals to identify the sources of any significant differences. Significance was determined at an alpha of 0.05.

When comparing the lithic assemblage from Fazenda Matos with other primate-flaked assemblages and known Plio-Pleistocene assemblages, a Principal Component Analysis (PCA) was conducted. This analysis used a range of measures related to the technology of each assemblage and employed successfully to differentiate between Lomekwian, Oldowan, and early Acheulean assemblages during the Plio-Pleistocene (*SI Appendix*, Table S1) (7, 10, 57).

Furthermore, we investigated the level of variability within specific attributes of the complete flake assemblages from Fazenda Matos (capuchins), Lobi Bay (macaques), and various Oldowan sites from the Plio-Pleistocene using a pairwise Brown Forsythe test. These differences are visually illustrated using the pheatmap package.

All statistical tests were conducted using the R statistical package v4.4.0 (73).

## Supplementary Material

Appendix 01 (PDF)

Movie S1. Examples of yellow breasted capuchins (*Sapajus xanthosternos*) nutcracking at Fazenda Matos.

Movie S2. 3D video of refit set 1.

Movie S3. 3D video of refit set 2.

Movie S4. 3D video of refit set 3.

## Data Availability

CSV files data have been deposited in OSF (doi.org/10.17605/OSF.IO/HDJ2V) (74).

## References

[1] J. E. Lewis, S. Harmand, An earlier origin for stone tool making: Implications for cognitive evolution and the transition to Homo. Philos. Trans. R. Soc. B Biol. Sci. **371**, 20150233 (2016). 10.1098/rstb.2015.0233 PMC4920290 27298464

[2] S. Harmand , 3.3-million-year-old stone tools from Lomekwi 3, West Turkana, Kenya. Nature **521**, 310–315 (2015). 25993961 10.1038/nature14464

[3] W. Archer, V. Aldeias, S. P. McPherron, What is ‘in situ’? A reply to Harmand et al. (2015). J. Hum. Evol. **142**, 102740 (2020). 32247106 10.1016/j.jhevol.2020.102740

[4] M. Domínguez-Rodrigo, L. Alcalá, Pliocene archaeology at Lomekwi 3? New evidence fuels more skepticism J. Afr. Archaeol. **17**, 173–176 (2019).

[5] S. McPherron, Evidence for stone-tool-assisted consumption of animal tissue 3.39 million years ago at Dikika, Ethiopia. Nature **466**, 857–860 (2010). 20703305 10.1038/nature09248

[6] M. Domínguez-Rodrigo, T. R. Pickering, H. T. Bunn, Reply to McPherron et al.: Doubting Dikika is about data, not paradigms. Proc. Natl. Acad. Sci. U.S.A. **108**, E117–E117 (2011).21536920

[7] T. W. Plummer , Expanded geographic distribution and dietary strategies of the earliest Oldowan hominins and Paranthropus. Science **379**, 561–566 (2023). 36758076 10.1126/science.abo7452

[8] I. de la Torre , New excavations in the MNK Skull site, and the last appearance of the Oldowan and Homo habilis at Olduvai Gorge, Tanzania. J. Anthropol. Archaeol. **61**, 101255 (2021).

[9] M. D. Leakey, Olduvai Gorge: Volume 3, Excavations in Beds I and II, 1960–1963. (Cambridge University Press, 1971).

[10] D. R. Braun , Earliest known Oldowan artifacts at >2.58 Ma from Ledi-Geraru, Ethiopia, highlight early technological diversity. Proc. Natl. Acad. Sci. U.S.A. **116**, 11712–11717 (2019). 31160451 10.1073/pnas.1820177116 PMC6575601

[11] S. Prat, Beyond the genus stereotype. Who were the first toolmarkers in Africa? Crossed views between archaeology and anatomy L’Anthropologie **127**, 103187 (2023).

[12] R. Susman, Who made the Oldowan tools? Fossil evidence for tool behaviour in Plio-Pleistocene hominids J. Anthropol. Res. **47**, 129–151 (1991).

[13] S. Prat, Beyond the genus stereotype. Who were the first toolmarkers in Africa? Crossed views between archaeology and anatomy L’Anthropologie **127**, 103187 (2023).

[14] C. J. Lepre , An earlier origin for the Acheulian. Nature **477**, 82–85 (2011). 21886161 10.1038/nature10372

[15] J. D. Clark, Kalambo Falls Prehistoric Site: Volume 3, The Earlier Cultures: Middle and Earlier Stone Age. (Cambridge University Press, 1969).

[16] A. L. Deino, S. McBrearty, 40Ar/39Ar dating of the Kapthurin Formation, Baringo, Kenya. J. Hum. Evol. **42**, 185–210 (2002). 11795974 10.1006/jhev.2001.0517

[17] I. De La Torre, The origins of the Acheulean: Past and present perspectives on a major transition in human evolution. Philos. Trans. R. Soc. B **371**, 20150245 (2016). 10.1098/rstb.2015.0245 PMC4920301 27298475

[18] S. Carvalho, M. Beardmore-Herd, “Technological origins: Primate perspectives and early hominin tool use in Africa” in Oxford Research Encyclopedia of African History (Oxford University Press, 2019).

[19] C. Rolian, S. Carvalho, “Tool use and manufacture in the last common ancestor of Pan and Homo” in Chimpanzees and Human Evolution, M. N. Muller, R. W. Wrangham, D. Pilbeam, Eds. (Harvard University Press, 2017), pp. 602–644.

[20] L. F. Marchant, W. C. McGrew, “Percussive technology: Chimpanzee baobab smashing and the evolutionary modeling of hominid knapping” in Stone Knapping: The Necessary Conditions for a Uniquely Hominid Behavior. Cambridge: McDonald Institute for Archaeological Research. P. V. Roux, B. Bril, Eds. (McDonald Institute Monographs, 2005), pp. 341–352.

[21] J. C. Thompson, S. Carvalho, C. W. Marean, Z. Alemseged, Origins of the human predatory pattern: The transition to large-animal exploitation by early hominins. Curr. Anthropol. **60**, 1–23 (2019).

[22] L. V. Luncz, A. Arroyo, T. Falótico, P. Quinn, T. Proffitt, A primate model for the origin of flake technology. J. Hum. Evol. **171**, 103250 (2022). 36122461 10.1016/j.jhevol.2022.103250

[23] S. Almécija, S. Moyà-Solà, D. M. Alba, Early origin for human-like precision grasping: A comparative study of pollical distal phalanges in fossil hominins. PLOS ONE **5**, e11727 (2010). 20661444 10.1371/journal.pone.0011727 PMC2908684

[24] M. M. Skinner , Human-like hand use in Australopithecus africanus. Science **347**, 395–399 (2015). 25613885 10.1126/science.1261735

[25] I. de la Torre, The origins of stone tool technology in Africa: A historical perspective. Philos. Trans. R. Soc. Lond. B Biol. Sci. **366**, 1028–1037 (2011). 21357225 10.1098/rstb.2010.0350 PMC3049100

[26] A. C. Hannah, W. C. McGrew, Chimpanzees using stones to crack open oil palm nuts in Liberia. Primates **28**, 31–46 (1987).

[27] R. B. Gürbüz, S. J. Lycett, Did the use of bone flakes precede the use of knapped stone flakes in hominin meat processing and could this be detectable archaeologically? J. Anthropol. Archaeol. **62**, 101305 (2021).

[28] T. Proffitt , Wild monkeys flake stone tools. Nature **539**, 85–88 (2016). 27760117 10.1038/nature20112

[29] T. Proffitt, J. S. Reeves, D. R. Braun, S. Malaivijitnond, L. V. Luncz, Wild macaques challenge the origin of intentional tool production. Sci. Adv. **9**, eade8159 (2023). 36897944 10.1126/sciadv.ade8159 PMC10005173

[30] T. Proffitt, M. Haslam, J. F. Mercader, C. Boesch, L. V. Luncz, Revisiting Panda 100, the first archaeological chimpanzee nut-cracking site. J. Hum. Evol. **124**, 117–139 (2018). 30236627 10.1016/j.jhevol.2018.04.016

[31] S. Carvalho, E. Cunha, C. Sousa, T. Matsuzawa, Chaînes opératoires and resource-exploitation strategies in chimpanzee (Pan troglodytes) nut cracking. J. Hum. Evol. **55**, 148–163 (2008). 18359504 10.1016/j.jhevol.2008.02.005

[32] B. J. Barrett , Habitual stone-tool-aided extractive foraging in white-faced capuchins, Cebus capucinus. R. Soc. Open Sci. **5**, 181002 (2018). 30225086 10.1098/rsos.181002 PMC6124021

[33] A. Whiten , Cultures in chimpanzees. Nature **399**, 682–685 (1999). 10385119 10.1038/21415

[34] T. Falótico, E. B. Ottoni, The manifold use of pounding stone tools by wild capuchin monkeys of Serra da Capivara National Park. Brazil. **153**, 421–442 (2016).

[35] M. D. Gumert, S. Malaivijitnond, Marine prey processed with stone tools by Burmese long-tailed macaques (Macaca fascicularis aurea) in intertidal habitats. Am. J. Phys. Anthropol. **149**, 447–457 (2012). 23042618 10.1002/ajpa.22143

[36] L. V. Luncz , Technological response of wild macaques (Macaca fascicularis) to anthropogenic change. Int. J. Primatol. **38**, 872–880 (2017). 29056799 10.1007/s10764-017-9985-6 PMC5629225

[37] J. Reeves, S. T. Proffitt, L.V. Luncz, Modeling a primate technological niche. Sci. Rep. **11**, 23139 (2021). 34848740 10.1038/s41598-021-01849-4 PMC8632893

[38] T. Falótico, T. Proffitt, E. B. Ottoni, R. A. Staff, M. Haslam, Three thousand years of wild capuchin stone tool use. Nat. Ecol. Evol. **3**, 1034–1038 (2019). 31235926 10.1038/s41559-019-0904-4

[39] M. Haslam , Archaeological excavation of wild macaque stone tools. J. Hum. Evol. **96**, 134–138 (2016). 27256780 10.1016/j.jhevol.2016.05.002

[40] J. Mercader, 4,300-Year-old chimpanzee sites and the origins of percussive stone technology. Proc. Natl. Acad. Sci. U.S.A. **104**, 3043–3048 (2007). 17360606 10.1073/pnas.0607909104 PMC1805589

[41] M. Mannu, E. B. Ottoni, The enhanced tool-kit of two groups of wild bearded capuchin monkeys in the Caatinga: Tool making, associative use, and secondary tools. Am. J. Primatol. **71**, 242–251 (2009). 19051323 10.1002/ajp.20642

[42] G. R. Canale, C. E. Guidorizzi, M. C. M. Kierulff, C. A. F. R. Gatto, First record of tool use by wild populations of the yellow-breasted capuchin monkey (Cebus xanthosternos) and new records for the bearded capuchin (Cebus libidinosus). Am. J. Primatol. **71**, 366–372 (2009). 19206141 10.1002/ajp.20648

[43] A. Arroyo, S. Harmand, H. Roche, N. Taylor, Searching for hidden activities: Percussive tools from the Oldowan and Acheulean of West Turkana, Kenya (2.3–1.76 ma). J. Archaeol. Sci. **123**, 105238 (2020).

[44] A. Arroyo, I. de la Torre, Pounding tools in HWK EE and EF-HR (Olduvai Gorge, Tanzania): Percussive activities in the Oldowan-Acheulean transition. J. Hum. Evol. **120**, 402–421 (2018). 29169680 10.1016/j.jhevol.2017.10.005

[45] I. de la Torre, S. Hirata, Percussive technology in human evolution: An introduction to a comparative approach in fossil and living primates. Philos. Trans. R. Soc. B Biol. Sci. **370**, 20140346 (2015). 10.1098/rstb.2014.0346 PMC4614711 26483526

[46] I. de la Torre, R. Mora, A technological analysis of non-flaked stone tools in Olduvai Beds I & II. Stressing the relevance of percussion activities in the African Lower Pleistocene. PALEO. Rev. Archéol. Préhist. 13–34 (2010).

[47] I. de la Torre, R. Mora, Percussion tools in Olduvai Beds I and II (Tanzania): Implications for early human activities. J. Anthropol. Archaeol. **24**, 179–192 (2005).

[48] J. Chavaillon, Essai pour une typologie du matériel de percussion. Bull. Soc. Préhist. Fr. **76**, 230–233 (1979).

[49] T. Proffitt , Analysis of wild macaque stone tools used to crack oil palm nuts. R. Soc. Open Sci. **5**, 171904 (2018). 29657792 10.1098/rsos.171904 PMC5882716

[50] C. Boesch, H. Boesch, Optimisation of nut-cracking with natural hammers by wild chimpanzees. Behaviour **83**, 265–286 (1983).

[51] T. Falótico , Analysis of sea almond (Terminalia catappa) cracking sites used by wild Burmese long-tailed macaques (Macaca fascicularis aurea). Am. J. Primatol. **79**, e22629 (2017). 10.1002/ajp.22629 28056164

[52] M. D. Gumert, S. Malaivijitnond, Marine prey processed with stone tools by Burmese long-tailed macaques (Macaca fascicularis aurea) in intertidal habitats. Am. J. Phys. Anthropol. **149**, 447–457 (2012). 23042618 10.1002/ajpa.22143

[53] M. A. Panger, A. S. Brooks, B. G. Richmond, B. Wood, Older than the Oldowan? Rethinking the emergence of hominin tool use Evol. Anthropol. **11**, 235–245 (2002).

[54] I. de la Torre, Searching for the emergence of stone tool making in eastern Africa. Proc. Natl. Acad. Sci. U.S.A. **116**, 11567–11569 (2019). 31164417 10.1073/pnas.1906926116 PMC6575166

[55] J. S. Reeves, T. Proffitt, S. Malaivijitnond, L. V. Luncz, Emergent technological variation in archaeological landscapes: A primate perspective. J. R. Soc. Interface **20**, 20230118 (2023). 37340784 10.1098/rsif.2023.0118 PMC10282572

[56] C. Rolian, S. Carvalho, “Tool use and manufacture in the last common ancestor of Pan and Homo” in Chimpanzees and Human Evolution, M. N. Muller, R. W. Wrangham, D. Pilbeam, Eds. (Harvard University Press, 2017), pp. 602–644.

[57] E. M. Finestone , New Oldowan locality Sare-Abururu (ca. 1.7 Ma) provides evidence of diverse hominin behaviors on the Homa Peninsula, Kenya. J. Hum. Evol. **190**, 103498 (2024). 38581918 10.1016/j.jhevol.2024.103498

[58] S. Semaw, The world’s oldest stone artefacts from Gona, Ethiopia: Their implications for understanding stone technology and patterns of human evolution between 2.6–1.5 million years ago. J. Archaeol. Sci. **27**, 1197–1214 (2000).

[59] J. Mercader , Earliest Olduvai hominins exploited unstable environments ~2 million years ago. Nat. Commun. **12**, 3 (2021). 33414467 10.1038/s41467-020-20176-2 PMC7791053

[60] I. de la Torre, Omo revisited: Evaluating the technological skills of Pliocene hominids. Curr. Anthropol. **45**, 439–465 (2004).

[61] I. de la Torre, R. Mora, Technological Strategies in the Lower Pleistocene at Olduvai Beds I & II. (Etudes et Recherches Archeologiques de l’Universite de Liege, 2005).

[62] T. Proffitt, Is there a Developed Oldowan A at Olduvai Gorge? A diachronic analysis of the Oldowan in Bed I and Lower-Middle Bed II at Olduvai Gorge, Tanzania J. Hum. Evol. **120**, 92–113 (2018). 29752004 10.1016/j.jhevol.2018.01.006

[63] A. Delagnes, H. Roche, Late Pliocene hominid knapping skills: The case of Lokalalei 2C, West Turkana, Kenya. J. Hum. Evol. **48**, 435–472 (2005). 15857650 10.1016/j.jhevol.2004.12.005

[64] G. R. Canale , “Sapajus xanthosternos (amended version of 2020 assessment)” in IUCN Red List Threat Species (2021).

[65] M. C. M. Kierulff, G. Canale, P. S. Gouveia, Monitoring the yellow-breasted capuchin monkey (Cebus xanthosternos) with radiotelemetry: Choosing the best radiocollar. Neotrop. Primates **13**, 32–33 (2005).

[66] I. de la Torre, R. Mora, Technological Strategies in the Lower Pleistocene at Olduvai Beds I & II. (Etudes et Recherches Archeologiques de l’Universite de Liege, 2005).

[67] I. de la Torre, A. Benito-Calvo, A. Arroyo, A. Zupancich, T. Proffitt, Experimental protocols for the study of battered stone anvils from Olduvai Gorge (Tanzania). J. Archaeol. Sci. **40**, 313–332 (2013).

[68] A. Arroyo, S. Hirata, T. Matsuzawa, I. de la Torre, Nut cracking tools used by captive chimpanzees (Pan troglodytes) and their comparison with early stone age percussive artefacts from Olduvai Gorge. PLoS ONE **11**, e0166788 (2016). 27870877 10.1371/journal.pone.0166788 PMC5117719

[69] J. Adams , “Functional analysis of macro-lithic artefacts. Non-flint raw material use in prehistory old prejudices and new direction” in 15th UISPP congress, Lisbon-September 2006, BAR International Series 1939. (2009).

[70] N. Toth, The Oldowan reassessed: A close look at early stone artifacts. J. Archaeol. Sci. **12**, 101–120 (1985).

[71] E. D. Sneed, R. L. Folk, Pebbles in the Lower Colorado River, Texas a study in particle morphogenesis. J. Geol. **66**, 114–150 (1958).

[72] Ž Režek, H. L. Dibble, S. P. McPherron, D. R. Braun, S. C. Lin, Two million years of flaking stone and the evolutionary efficiency of stone tool technology. Nat. Ecol. Evol. **2**, 628–633 (2018). 29507377 10.1038/s41559-018-0488-4

[73] R Core Team, R: A language and environment for statistical computing (R Foundation for Statistical Computing, Vienna, Austria, 2021). https://www.R-project.org/. Accessed 10 January 2024.

[74] T. Proffitt, P. de Sousa Medeiros, W. P. Martins, L. V. Luncz, Data from “Flake production: A universal by product of primate stone percussion.” OSF. 10.17605/OSF.IO/HDJ2V. Deposited 16 January 2025. PMC11848292 39933001

