# Supplementary material for: Flake production: A universal by-product of primate stone percussion
Source: Proc Natl Acad Sci U S A. 2025 Feb 11;122(7):e2420067122. doi: 10.1073/pnas.2420067122 (PMC11848292; doi:10.1073/pnas.2420067122)
Supplement: Supplementary file 1 — Appendix 01 (PDF) [file pnas.2420067122.sapp.pdf]

Supplementary Material

Flake Production: A Universal Byproduct of Primate Stone Percussion.

Tomos Proffitt* [1,2], Paula de Sousa Medeiros [3], Waldney Pereira Martins [3,4], Lydia. V. Luncz*[1]

[1]. Technological Primates Research Group, Max Planck Institute for Evolutionary Anthropology, Deutscher Platz 6, Leipzig, 04103, Germany

[2] Interdisciplinary Center for Archaeology and Evolution of Human Behaviour (ICArEHB), Universidade do Algarve, Faro, Portugal.

[3].Programa de Pós-Graduação em Biodiversidade e Uso dos Recursos Naturais, Universidade Estadual de Montes Claros, Montes Claros, MG, Brazil

[4]Departamento de Biologia Geral, Centro de Ciências Biológicas e da Saúde, Universidade Estadual de Montes Claros, Montes Claros, MG, Brazil

*Corresponding Author: tomos_proffitt@eva.mpg; tsproffitt@ualg.pt; lydia_luncz@eva.mpg.de

Supplementary Information

1. Lithic Analysis - Definition of terms

The lithic analysis follows definitions and protocols previously applied to other primate flaked lithic assemblages in (1–3). We provide a re-iteration of the technological definitions used and attributes analysed in this study below.

All material was classified broadly following Isaacs (4) classification of lithic material: Flaked pieces, detached pieces, pounded pieces, and unmodified / natural pieces. In the original definition, flaked pieces refer to intentionally flaked cores. However, in this study, the term refers to hammerstones with one or more flake detachments. Technological classifications were applied to flaked and detached pieces. Detached pieces were classified into complete flakes, fragmented flakes, and small debris (<20mm). Linear maximum dimensions (mm) and mass (g) were recorded for artefacts, with additional measurements recorded for complete flakes).

Hammerstones and broken hammerstones: Blank type (cobble, fragment, nodule, block), original blank morphology (rounded, tabular, plano-convex, irregular), number of utilized planes, percussive damage surface morphology (flat, convex, ridge), and degree of percussive damage (qualitatively assessed as light, medium and heavy) was recorded for all complete and broken hammerstones.

Flaked pieces: In addition to the percussive attributes listed above, the following technological attributes were also recorded for flaked pieces. The number of detachments greater than 10mm in maximum dimension along with their maximum linear dimensions. Flake initiation type (conchoidal, wedge, bending, indeterminate), core flaking accidents (step scars, plunging scars), degree of cortex coverage (0%, <50%, >50%), and number of extractions (n). Each flaked piece was also classified into reduction types following classifications described in de la Torre (5, 6) indicating the prevailing direction and angle of flake removals.

Complete Flakes: Complete flakes, defined as detached pieces, possessing ventral and dorsal surfaces separated by a sharp edge, and a complete platform and impact point. Flakes could be conchoidal, wedge or bending initiated following Andrefsky (7). In addition to linear maximum dimensions, additional measurements were recorded including maximum dimensions (length, width, thickness) (7) and technological dimensions (length and width) measured from the platform to the distal end and maximum measurement orthogonal to the technological length (8). These dimensional measures were used to calculate additional shape variables including elongation (technological length / technological width), flattening (thickness / technological width), area (maximum length x maximum width), and volume (maximum length x maximum width x maximum thickness). Platform depth and width were recorded and used to calculate platform area, and flattening. External platform angle (EPA) were recorded.

Technological attributes recorded follow those set out in Mora et al (9) and Proffitt (10) which include striking platform cortex (0%, <50%, >50% and 100%), platform morphology (platform, lineal, punctiform), platform faceting (non-faceted, uni-faceted, bi-faceted, multi- faceted), platform shape (flat, convex, concave, uni-angular, irregular), bulb of percussion (marked, diffuse, fractured, indeterminate), knapping accidents (step, hinge, plunging terminations), presence of dorsal surface step scars, transversal and sagittal cross section shape, dorsal surface cortex (measured the same as platform cortex), number of dorsal extractions, dorsal extraction directionality, and flake categories following Toth (11) ranging from I to VI representing early (I-III) and late (IV-VI) stages of reduction. Furthermore, percussion attributes were recorded, including: the presence or absence of dorsal surface and platform percussive damage, and the number of percussive impacts on the dorsal surface.

2. Lithic Analysis

General frequencies and raw materials:

Fazenda Matos falls within the Ediacaran Serra de Santa Helena Geological Formation, with an underlying geology of a combination of siltstones, ardosia, claystones and marls. Based on a visual identification, the raw material which makes up the lithic assemblages from Fazenda Matos is a homogeneous fine-grained siltstone which possesses a mean hardness value of 44.8 (min = min = 27, max = 91.5, sd = 7.6). The hardness values taken across the entire study region possess a small coefficient of variation (cv = 0.17) suggesting they are from the same source (SOM Fig S1).

Due to the isotopic nature and generally tabular morphologies of the available raw material at FM, capuchin nut cracking against stone anvils often results in a highly fragmented lithic assemblage (*n* = 357; SOM Table S1). Although not all technological categories are represented at each sampled nut cracking site, at a total assemblage level all categories are present (Figure 2; SOM Table S1). Detached pieces, including complete flakes, broken flakes, hammerstone flakes, small debris and angular chunks make up the majority of the assemblage by frequency (*n* = 333, 93.27%) (Figure 2; SOM Table S1). Formal tools (hammerstones, broken hammerstones, and flaked hammerstones) make up a substantially smaller proportion of the assemblage (n=24, 6.72%). In terms of total mass, however, the tools contribute substantially more of the total mass of stones in the assemblage (21.76kg, 86.49%) (Figure 2; SOM Table S1).

Hammerstones, fragmented hammerstones and flaked pieces:

Hammerstones in this assemblage can be separated into complete, broken and flaked categories. Complete hammerstones consist of non-broken or fractured tools with percussive damage on one or more of their active planes (Figure 3a-b). Broken hammerstones, exhibit one or more fractures through use whilst flaked hammerstones (flaked pieces) exhibit evidence of percussive damage through use as a hammerstone alongside one or more flake detachment. When the dimensions and mass of both complete hammerstones and flaked hammerstones are compared the available natural stones within the study area, there is no significant difference between the two samples (SOM Table S2). Considering both complete and flaked hammerstones they primarily fall within the bladed morphology (n = 7, 46.7%), with a small majority being classified as elongated (*n* = 4, 26.7%; Fig S2), A Chi-Square test (χ^2 = 10.353, df = 9, *p* = 0.323) shows no significant difference between hammerstone morphology and natural raw material distribution in the study area (SOM Fig S2; SOM Table S2).

Only two hammerstones in this assemblage can be considered complete (SOM Table S1), with no evidence of fracture or flake detachments. On average they measure 130.6 x 73.4 x 53.2 and possess a mean mass of 815.8 g (SOM Table S3). They possess percussive damage across multiple active planes, ranging from two to six, and located on both ridges, flat and convex surfaces. Regardless of the location, the damage is broadly the same, comprising of superimposed impact points and varying levels of crushing of the cortical surfaces often resulting in crushing of the edges.

Nine hammerstones can be classified as broken or fragmented due to use (SOM Table S1). They possess mean dimensions of 97.9.6 x 68.2 x 41.5 mm and a mean mass of 335.8g (SOM Table S3). Similarly, however, to complete hammerstones, they possess either clustered (*n* = 4, 44.4%) dispersed (*n* = 4, 44.4%) or isolated (n=1, 11.1%) percussive damage characterised by impact points (*n* = 9, 100%) and crushing (*n* = 6, 66.7%) along ridges (*n* = 7, 77.7%) and on flat (*n* = 6, 66.6%) surfaces. Most broken hammerstones possess a single breakage caused through use (*n* = 5, 55.5%), however, some have fragmented to a greater extent with >3 fractures (*n*= 4, 44.4%).

Although flaked pieces constitute a relatively small frequency, they are by far the heaviest technological group, accounting for 68% of the total mass of stones within the assemblage (SOM Table S1). They possess a mean length, width, and thickness of 148.8 mm x 94.8 mm x 20.9 mm with a mean mass of 254 g but have been

documented as heavy as 780.3g (SOM Table S3). There is no significant difference in length (W = 21, p = 0.228), thickness (W = 15, p = 0.8) nor mass (W = 20, p = 0.305) of flaked pieces compared to complete hammerstones within this assemblage, however, they are significantly wider (W= 25, p = 0.038).

These hammerstones, through heavy use, have resulted in the detachment of one or more flakes. Whilst these artifacts have been classified as flaked pieces, their primary function were as active hammerstones as opposed to flake cores. Nonetheless, they possess many of the same technological attributes typically associated with archaeological flaked pieces.

All flaked pieces exhibit either tabular or plano-convex morphology and feature multiple natural 90-degree or acute angles between the active and adjoining planes (SOM Table S4). Percussive damage is extensive on all flaked pieces and is often present across multiple active surfaces. In most cases, however, percussive damage is located on two flat opposed planes. The widespread nature of the percussive damage on these artifacts indicates their function as hammerstones and suggests a frequent rotation of the active surfaces during use, but with a preference for the larger flat surfaces.

The active surfaces in conjunction with frequent naturally acute edge angles serve as effective platforms for detachments on adjoining surfaces. A total of 119 complete flake scars with at least one maximum dimension greater than 10mm were counted on all flaked pieces, resulting in an average of 9.15 flake scars. However, in some cases the number of flake scars is considerably higher, or lower (SOM Table S5). On average, flake scars possess a length and width of 27.9 x 39.6 mm and a mean elongation value of 0.75 (min = 0.29, max = 2.13, SD = 0.34) (SOM Table S5) indicating a general trend of wide and short flake detachments, however, some instances of more elongated detachments are present (SOM Table S6). Furthermore, there are instances of flake scars possessing a maximum length or width in excess of 50mm, indicating, that in some cases nut cracking can result in relatively large detachments (SOM Fig S3).

All flaked pieces have developed varying degrees of percussive damage and edge battering (n = 11, 84.6%), often across multiple active surfaces. Where these active surfaces intersect with adjoining surfaces at angles of less than 90 degrees, there is almost always at least one flake detachment present. In many cases, these flake detachments are contiguous along a portion of the edge and superimposed, resulting in multiple overlapping unidirectional flake scars. These are often invasive across the length of the flaking surface (Figure 3c-f).

Based on the location and direction of flake scars flaked pieces can be separated into various exploitation patterns. The majority of these are unifacial and unidirectional exploitation (n = 10, 77%), however, both bifacial (n = 2, 15.4%) and multifacial (n = 1, 7.69%) exploitation are also present within the assemblage (SOM Table S4). Unifacial abrupt exploitation on one or two planes, where the angle between the flaking surface and the platform is >45° are most prevalent (n = 6, 46.2%), however, unifacial simple reduction (where the angle between the flaking surface and platform is <45°) is also represented (n = 4, 30.8%). Two flaked pieces exhibit a bifacial simple reduction pattern, where flakes have been detached from two adject platforms with the angle between them is <45°. Finally, a single flaked piece has been reduced multifacially, attesting to the frequent rotation of the hammerstones during use (SOM Table S4).

Complete Flakes:

When all complete flakes from each assemblage are combined, they possess an average maximum length, width, and thickness of 39.7 x 25 x 8.9 mm, with an average mass of 15.8 grams (SOM Table S3). Generally, these percussive flakes tend to be wide and short, displaying mean technological dimensions of 27.7 x 37.5 mm and a mean elongation ratio of 0.81 (min = 0.32, max = 2.23, sd = 0.35). Considerably larger and more elongated flakes are, however, also present within the collection (SOM Fig S4).

Detached flakes, possess a mean exterior platform angle of 91.9° (min = 65°, max = 134°, sd = 15.3°). Additionally, a variety of bulb morphologies are represented, including diffused (n = 53, 57.6%) and marked (n = 14, 15.2%) bulbs, as well as instances where bulbs are entirely absent (n = 23, 25%). Consequently, it can be argued that this assemblage comprises a mixture of conchoidal and wedge-initiated detachments.

On average, platforms on flakes tend to be relatively large, measuring 24.1 x 7.2 mm. The majority of impact points are centrally located (*n* = 55, 59.8%), with both cortical and rectilinear platforms being predominant (SOM Table S6). However, a small proportion of flakes possess platforms that are either entirely non-cortical or have had a degree of cortex removed (SOM Table S6), indicative of the occasional extended reduction sequences observed on the flaked pieces. Despite the prevalence of larger rectilinear platforms on most flakes (*n* = 75, 81.5%), indicating impacts away from the core edges, some flakes, however, possess, thinner lineal platforms (*n* = 16, 17.4%), indicating detachments from directly along the edges. When platforms lack cortex or have some cortex removed, the majority are uni-faceted (*n* = 8, 8.7%). However, instances of both bi-faceted (*n*=1, 1.09%) and multi-faceted (*n* = 2, 2.17%) platforms are also present, albeit in much smaller frequencies. Most platforms are flat (*n* = 81, 88%), although some are convex (*n* = 9, 9.78%) or uni-angular (*n*=2, 2.17%) in shape (SOM Table S6). These patterns of platform shape and preparation are consistent with the simple and ad-hoc nature of the detachment process for these flakes.

Most flakes possess feather terminations (*n* = 62, 67.4%), however, hinge, plunging and step terminations (SOM Table S6). The presence of these terminations indicates the variable force of the impact between hammerstone and anvil, as well as the absence of flaking surface management. Although step scars are present on the dorsal surface of multiple flakes, they are not ubiquitous (*n* = 32, 34.8%). Among these, the majority consist of single instances of step scars rather than stacks of scars (SOM Table S6).

While some flakes display fully cortical dorsal surfaces (*n* = 30, 32.6%), the majority either exhibit a degree of decortication (*n* = 40, 43.5%) or lack cortex entirely (*n* = 22, 23.9%). Overall, however, the proportion of flake categories indicates that this assemblage is dominated by the early stages of reduction, with the majority of flakes falling into stages I, II and III (*n* = 81, 88.1%) (SOM Table S6). There is a general lack of percussive damage present on the dorsal surfaces of flakes, with only 23.9% (*n* = 22) of the flake assemblage possessing percussive evidence indicative of being derived form a percussive behaviour. Among the flakes that possess dorsal scars the majority possess between two and five flake scars (*n* = 39, 62.9%), while the rest possess only flake scar. The presence of multiple scars on dorsal surfaces indicates a recurring pattern of detachments within the assemblage. These are by and large unidirectional (*n* = 52, 88.1%), with considerably smaller frequencies of opposed (*n* = 5, 8.5%) and transversal (*n* = 2, 3.4%) flake scar directionality (Figure 4a-f; SOM Fig S6).

Retouched piece:

One relatively large cortical flake exhibits clear evidence of percussive damage on its dorsal and ventral surface, indicating its use as a percussive tool following its detachment from a hammerstone or anvil. This damage is characterised as a small cluster of impact points located on a raised area of the ventral surface. Additionally, as a result of its use as a percussive tool, three short, contiguous non-invasive removals have been detached from its distal right edge, each showing distinct impact points and feather terminations (Figure 4g). Although the production of this object was not directly observed, its location away from known waterways and channels, combined with the absence of visible taphonomic disturbances such as trampling, suggests that this damage was produced as a consequence of reuse by capuchins monkeys as a small percussive tool.

Material signature comparison across species:

When comparing the two assemblages in their entirety, which includes the low-quality raw material from Lobi Bay, a clear difference in the frequency of technological categories between Fazenda Matos and Lobi Bay is present. The results of a chi-square test indicate that Fazenda Matos displays significantly fewer angular pieces, both with and without percussive damage, as well as fewer hammerstones. Conversely, it exhibits significantly higher frequencies of complete flakes, fragmented flakes, hammerstone flakes, and small debris (χ^2 = 343.53.701, df =9, p = <0.001; SOM Table S7).

However, once the low-quality limestone from Lobi Bay is excluded from the comparison, the variation between the two assemblages' shifts. While a significant difference persists (χ^2 = 34.701, df = 9, p < 0.001), it is primarily attributed to a higher frequency of complete hammerstones at Lobi Bay, in contrast to the

underrepresentation of hammerstones in the Fazenda Matos assemblage (SOM Table S7). Despite this variation, overall, the two assemblages maintain remarkable similarity in the relative frequency of different technological categories within each (Figure 6a-b).

Flaked Hammerstone Comparison:

Flaked pieces from Fazenda Matos exhibit significant differences in their size and weight compared to those from Lobi (Fig 6; SOM Table S8). They are consistently larger in all dimensions and heavier (SOM Fig S7). However, there is no significant variance in the degree of elongation or flattening between the two assemblages (Figure 6c-d; SOM Table S8).

While the Fazenda Matos pieces show a higher prevalence of step scars (SOM Table S9-10), the overall technological characteristics are quite similar between the two collections (Figure 6c-d). Both collections exhibit comparable levels of cortical coverage, and the number and patterns of flake removals (Figure 6d). Exploitation strategies, including the dominance of unifacial patterns and the limited presence of bifacial and multifacial patterns of removals, are consistent across both assemblages (SOM Table S10).

Comparison of complete flakes:

There is a significant difference in the dimensions of complete flakes between the two assemblages (Figure 6e). Flakes from Fazenda Matos are substantially larger both in length and width (both maximum and technological) and heavier. Furthermore, flakes from Fazenda Matos possess wider and larger platforms with significantly greater EPA values compared to Lobi Bay, however, platform depth remains similar between the assemblages (Figure 6e; SOM Table S11)).

Of the 14 technological attributes identified on complete flakes, only four showed significant differences between the two assemblages. These include the degree of dorsal cortex and the representation of reduction stages (Toth categories), the type of platform and the presence and quantity of step scars (Figure 6f; SOM Table S10).

The difference in reduction stage representation between the assemblages is driven by an increased frequency of stage III flakes in the Fazenda Matos flakes assemblages, with a concurrent increase in stage II and a slight rise in stage V flakes in the Lobi Bay assemblages (SOM Table S12). However, neither assemblage exhibit high levels of reduction, with no significant difference in number of dorsal scars present (SOM Table S10).

Whilst the flake assemblage from Fazenda Matos possesses a greater frequency of lineal platforms compared to more punctiform platforms at Lobi Bay (Fig 6f; SOM Table S12), the majority of the flakes from both assemblages possess clear platforms. Despite this, there is a striking similarity in the flake platforms between the two assemblages, with no significant differences in the representation of platform shapes, the amount of cortex, impact point location, and degree of platform faceting (Fig S6f; SOM Table S12-S13). In both assemblages, platforms tend to be cortical and as such non-faceted, relatively flat and with impact points located towards the centre.

Flakes from Fazenda Matos possess significantly more step scars on their dorsal surfaces (Fig S6f; SOM Table S12-S13) compared to Lobi Bay, mirroring the increased frequency of step scars on flaked pieces. This difference may be associated with variation in the manner and kinematics employed in hammerstone use between the two populations.

Both assemblages are primarily composed of flakes featuring cortical, flat platforms, with relatively few knapping accidents. However, Fazenda Matos exhibits a slightly higher frequency of hinge-terminating

flakes. Overall, both collections display diffuse bulbs of percussion and relatively flat or concave ventral surfaces.

Comparison with the archaeological record:

A PCA analysis of the technological features of Lomekwian, Oldowan and Early Acheulean, and the primate assemblages from Lobi Bay, Fazenda Matos and the bearded capuchin stone on stone percussive assemblage from Serra da Capivara National Park (SCNP) indicates that both nut cracking assemblages (yellow breasted capuchins and long-tailed macaques) are more closely aligned with Oldowan and Acheulean assemblages compared to Lomekwian and the stone on stone assemblage from SCNP (Figure 7a-b; SOM Table S14). While the assemblage from Lobi Bay falls within the range of variation for the Oldowan, the assemblage from Fazenda Matos falls within the range of Early Acheulean assemblages in the comparative sample. These are differentiated from the Oldowan assemblages by the flake scar-to-core size ratio, mean flake scar count, and core size (Figure 7b).

When considering only the Oldowan samples greater than 2 Ma, the Lomekwian site, and the primate assemblages, there exists a high degree of variation with these assemblages occupying notably different regions of the PCA plot (Figure 7c-d).

A pairwise Brown-Forsythe test indicates significant flake length variance across all comparisons, indicating clear distinctions between primate and Oldowan flake assemblages (SOM Table S15, SOM Fig S8). However, primate flakes are not consistently larger or smaller than Oldowan flakes. Post hoc Dunn's test results show that Fazenda Matos flakes are significantly longer than both Lobi Bay and Oldowan >2Ma flakes, while Lobi Bay flakes are significantly than all other groups (SOM Table S16, SOM Fig S8).

Variation in flake width is consistent across all groups, with no significant differences observed (SOM Table S15, SOM Fig S8). However, a post hoc Dunn's test indicates that Fazenda Matos flakes are significantly narrower than all Oldowan flake groups, and Lobi Bay flakes are significantly narrower compared to Oldowan <2Ma flakes (SOM Table S16, SOM Fig S8). While variation in thickness shows limited differences between groups, with a significant difference in variance only between Oldowan >2Ma and the Fazenda Matos flakes (SOM Table S15, SOM Fig S8) a post hoc Dunn's test indicates that all primate flakes are significantly thinner than all Oldowan flakes (SOM Table S16, SOM Fig S8).

Flake area shows significantly greater variability in two cases: Oldowan >2Mya vs Fazenda Matos flakes and Oldowan <2Mya vs Lobi Bay flakes (SOM Table S15, SOM Fig S8) . However, Fazenda Matos flakes have significantly smaller areas than Oldowan <2Ma flakes, while Lobi Bay flakes are significantly smaller than both Oldowan flake groups. The same patterns are observed for flake volume (SOM Table S16, SOM Fig S8).

Lobi Bay flakes exhibit significantly greater variance in mass compared to all Oldowan flakes, but this difference is not observed between Fazenda Matos and Oldowan flakes (SOM Table S15, SOM Fig S8). In all cases, primate flakes are significantly lighter than Oldowan flakes from both before and after 2Ma (SOM Table S16, SOM Fig S8).

Primate flake platform widths show greater variability than Oldowan flakes, with significant differences observed between the Fazenda Matos flakes and both Oldowan groups, as well as between the Lobi Bay flakes and Oldowan >2Ma flakes (SOM Table S15, SOM Fig S8). A post hoc Dunn's test shows that Fazenda Matos flakes have significantly wider platforms than Oldowan >2Ma flakes, while Lobi Bay flakes have significantly narrower platforms compared to Oldowan <2Ma flakes (SOM Table S16, SOM Fig S8). Platform depths shows significant differences in variability only between Fazenda Matos and Oldowan flakes (SOM Table S15, SOM Fig S8). However, metrically, only Lobi Bay flakes are significantly smaller in platform depth compared to Oldowan flakes (SOM Table S16, SOM Fig S8).

Similarly, platform area shows significant differences in variance only between Fazenda Matos and Oldowan flakes (SOM Table S15, SOM Fig S8). Metrically, only Lobi Bay flakes have significantly smaller platform areas compared to Oldowan flakes (SOM Table S16, SOM Fig S8). Conversely platform flattening is significantly more variable in both primate flake groups compared to Oldowan flakes (SOM Table S15, SOM Fig S8). Fazenda Matos flakes have significantly higher platform flattening values than both Oldowan flake groups, while Lobi Bay flakes have significantly higher values than Oldowan flakes older than 2Ma only (SOM Table S16, SOM Fig S8).

Exterior platform angles (EPA) are significantly more variable in all primate flake assemblages compared to both Oldowan flake groups and are also significantly larger than those of Oldowan flakes (SOM Table S15 -S16, SOM Fig S8).

Finally, variation in elongation and flake flattening is generally similar across the groups. However Lobi Bay flakes show significantly greater variability in flake flattening ratios compared to Oldowan flakes >2Ma (SOM Table S15-S16, SOM Fig S8).

Technological attributes of flakes show considerable variation between primate and Oldowan flakes. A chi-square test reveals significant differences between the two groups in the number of platform facets, dorsal scars, directions of dorsal scars, and the degree of dorsal cortex (SOM Table S17). Adjusted residuals indicate that primate flakes are significantly more likely to lack platform faceting compared to Oldowan flakes, both older and younger than 2 Ma (SOM Table S18, SOM Figure S9a). Additionally, primate flakes exhibit a higher frequency of flakes with no dorsal scars (SOM Table S18, SOM Figure S9b). Fazenda Matos flakes, in particular, show significantly less diverse dorsal scar patterns than Oldowan flakes older than 2 Ma (SOM Table S18, SOM Figure S9c). Lastly, primate flakes are significantly more likely to retain 100% dorsal cortex compared to Oldowan flakes (SOM Table S18, SOM Figure S9d).

When examining flaked pieces, significant differences in exploitation patterns are evident between primate and Oldowan assemblages ($X^2 = 153.63$, df = 52, $p < 0.001$). Adjusted residuals indicate that these differences stem from primate assemblages predominantly featuring unifacial exploitation strategies, while Oldowan assemblages exhibit both bifacial and multifacial strategies (SOM Figure S10; SOM Table S19-S20). The Fazenda Matos assemblages show a significant overrepresentation of unifacial abrupt unidirectional exploitation on two planes. In contrast, no significant over- or underrepresentation of exploitation patterns is observed in the Lobi Bay assemblage. The Lomekwi assemblage possesses a significant overrepresentation of unifacial abrupt bidirectional exploitation. Oldowan assemblages older than 2 Ma exhibit a significant overrepresentation of bifacial exploitation strategies, including bifacial simple, abrupt partial, peripheral, and polyhedral exploitation. These assemblages also show a significant underrepresentation of unifacial simple partial and abrupt exploitation patterns. Conversely, Oldowan assemblages younger than 2 Ma are significantly associated with unifacial centripetal exploitation, bifacial alternate total exploitation, and multifacial exploitation patterns.

Finally, flaked pieces associated with primate assemblages (Lobi Bay) possess a significant overrepresentation of single flaking surfaces, whilst Oldowan flaked pieces possess significantly greater frequencies of two (Oldowan >2Ma) or more (Oldowan <2Ma) flaking surface (SOM Figure S10; SOM Table S19-S20)

3. Refit Analysis

Refit 1

A large plano convex cobble used as a hammerstone with two active opposed planes, one being flat (Plane A) with the other being convex (Plane A2). A total of 40 detachments are present across this core, across 4 separate cortical flaking surfaces (Plane B, Plane C, Plane C2, and Plane B2). All detachments (apart from

three) originate from Plane A which acts as a cortical platform. All flaking surfaces intersect with the platform at a naturally acute angle.

Flake detachments early in the removal sequence tend to be large and invasive, often reaching beyond the midline of the core and resulting in the maintaining of an acute angle between the platform and flaking surfaces. The large early-stage detachments are followed by a series of smaller unidirectional superimposed detachments, often terminating in step fractures. There is little battering associated with impact points on Plane A, with the majority of the percussive damage located towards the centre of the plane. Thee small flake scars are also present on the platform (Plane A) originating form Plane C and Plane C, all of which possess step terminations (SOM Video S2).

Refit Set 2

A tabular cobble with two opposed percussive planes (Plane A and A2), both of which intersect with all other planes at naturally acute angles. Both plane have extensive percussive damage located towards their centre. A total of 17 detachments from two sequences separated by a rotation of the core are present on this refit. The first sequence uses plane A as a cortical platform resulting in 10 unidirectional superimposed removals from Planes C and C2. This is followed by a rotation of the core, with Plane A2 used as a platform resulting in the detachment of a further seven unidirectional superimposed removals from the centre of Plane B. There is a lack of battering associated with the impact points on both platforms (SOM Video S3).

Refit Set 3

A large rounded cobble used as a hammerstone as possessing a substantial amount of percussive damage across all planes derived from frequent rotation of the hammerstone during use. Due to excessive battering form use, two large unidirectional invasive detachments (one complete flake and one broken flake) are removed from Plane B (SOM Video S4).

Supplementary Figures

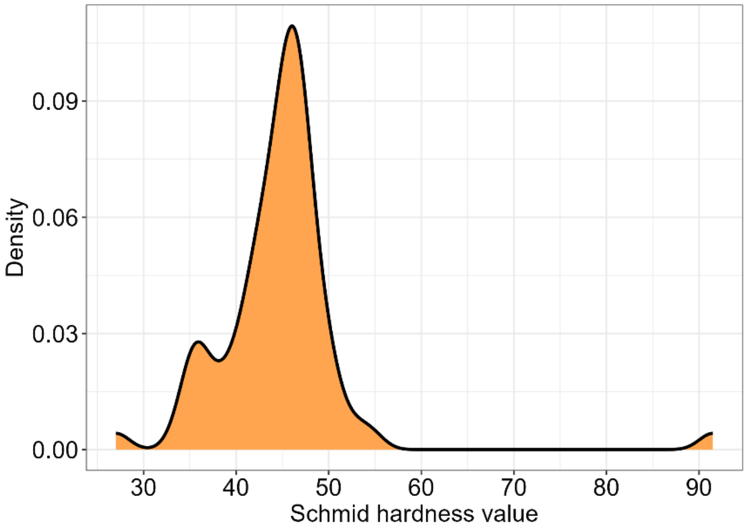

SOM Figure S1: Density plot showing the distribution of Schmidt hardness value for all sampled siltstone within the study region.

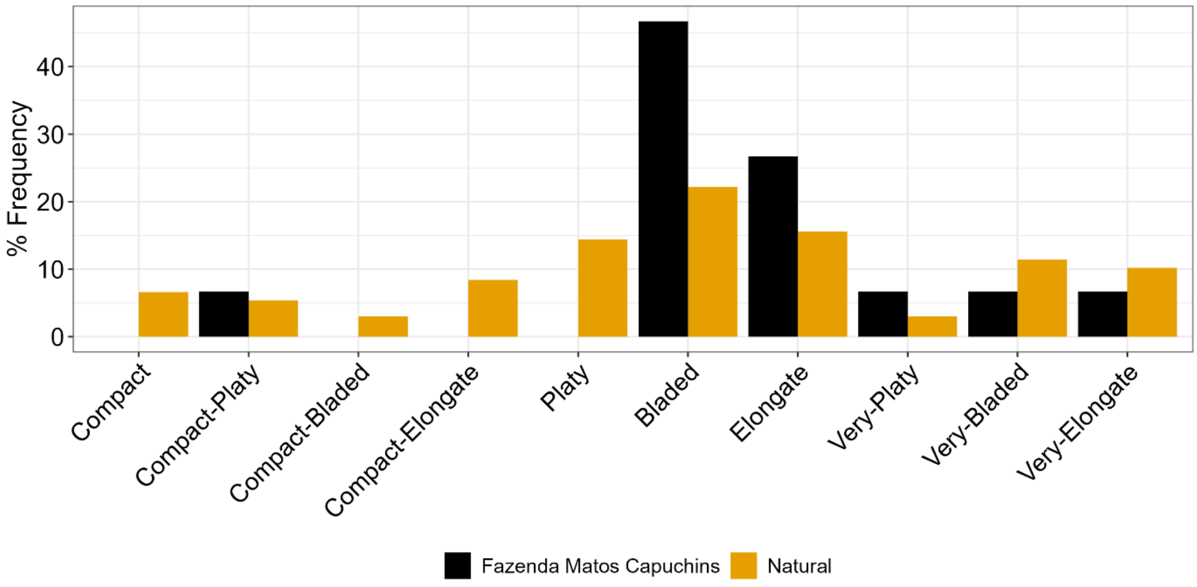

SOM Figure S2: Relative frequencies of hammerstone and natural stone morphologies at Fazenda Matos.

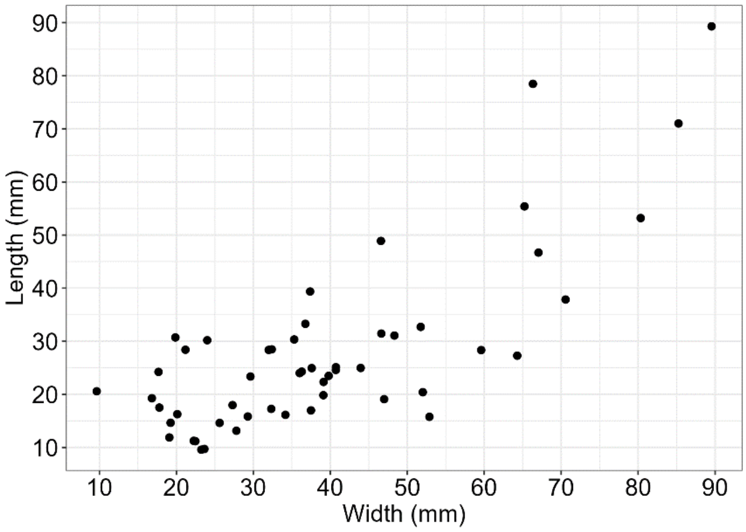

SOM Figure S3: Scatter plot showing the length and width (mm) distribution of all flake scars on flaked pieces at Fazenda Matos.

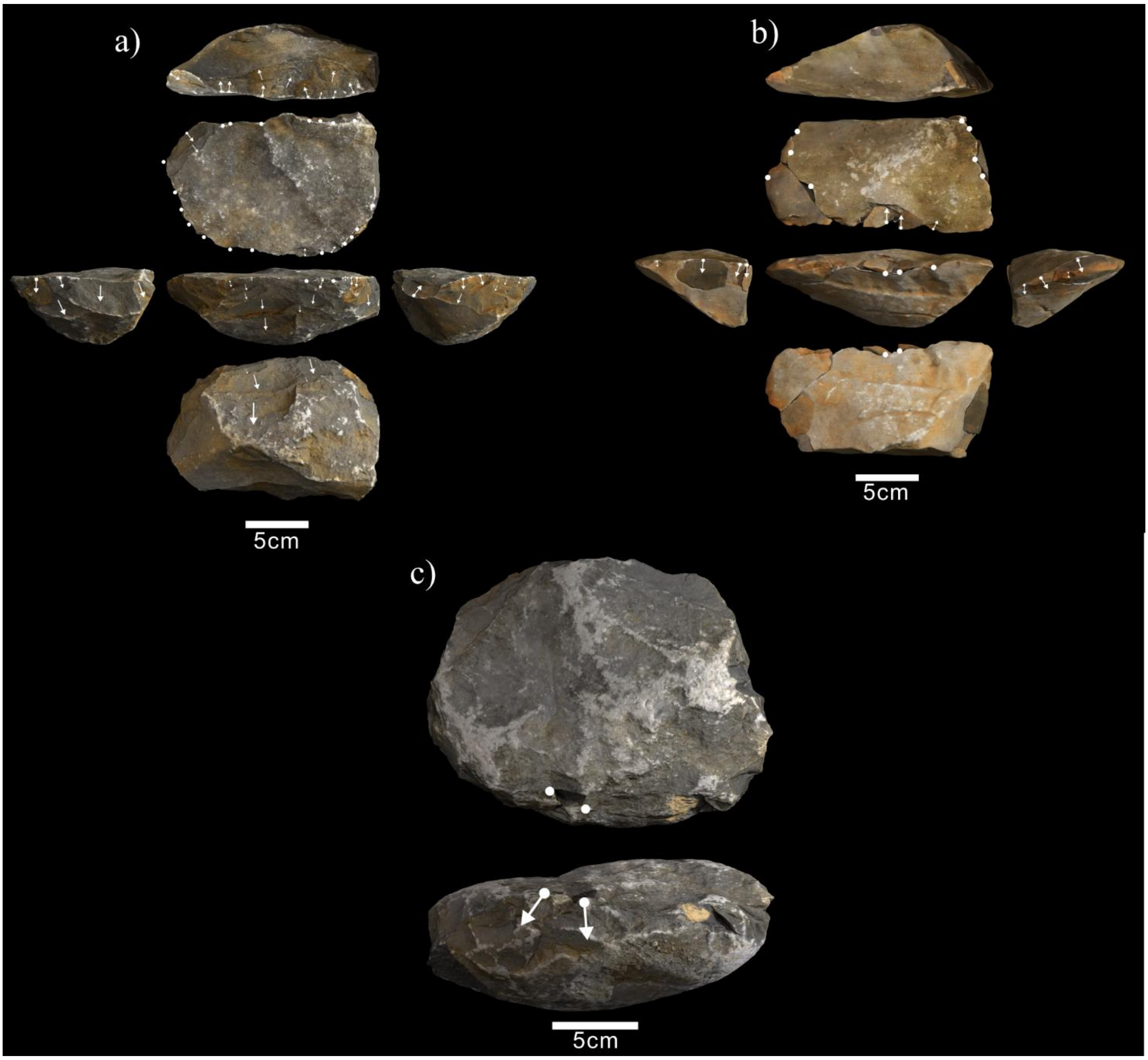

SOM Figure S4: Diacritic schema for three refits identified in the Fazenda Matos lithic assemblage.

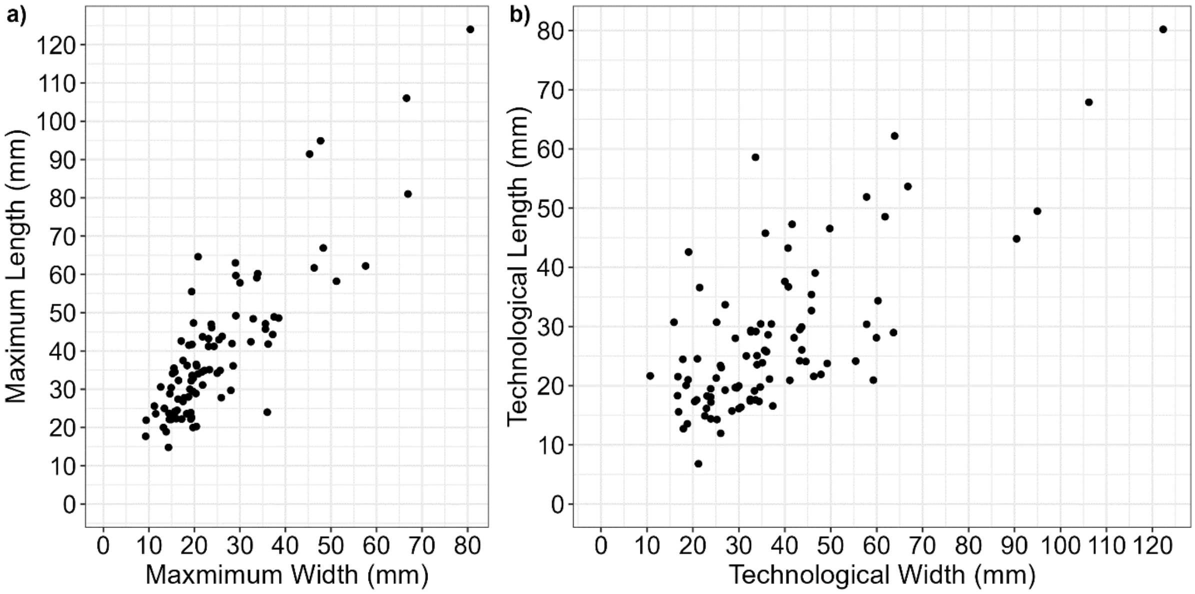

SOM Figure S5: Scatter plots showing the maximum (a) and technological (b) dimensions of all complete flakes.

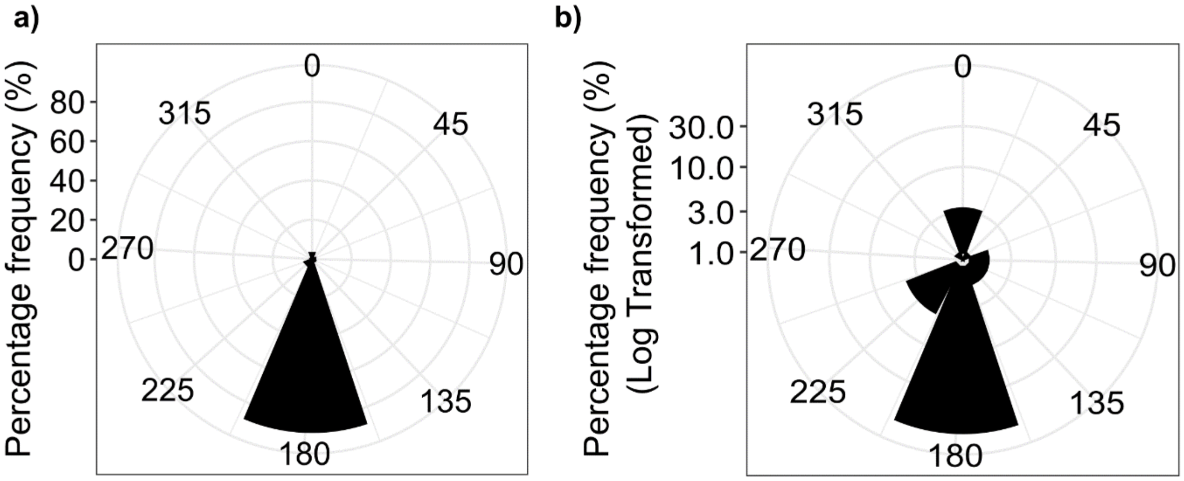

SOM Figure S6: Rose diagram illustrating the directionality of dorsal flake scars on complete flakes from Fazenda Matos, showing both relative frequencies (a) and relative frequencies log transformed

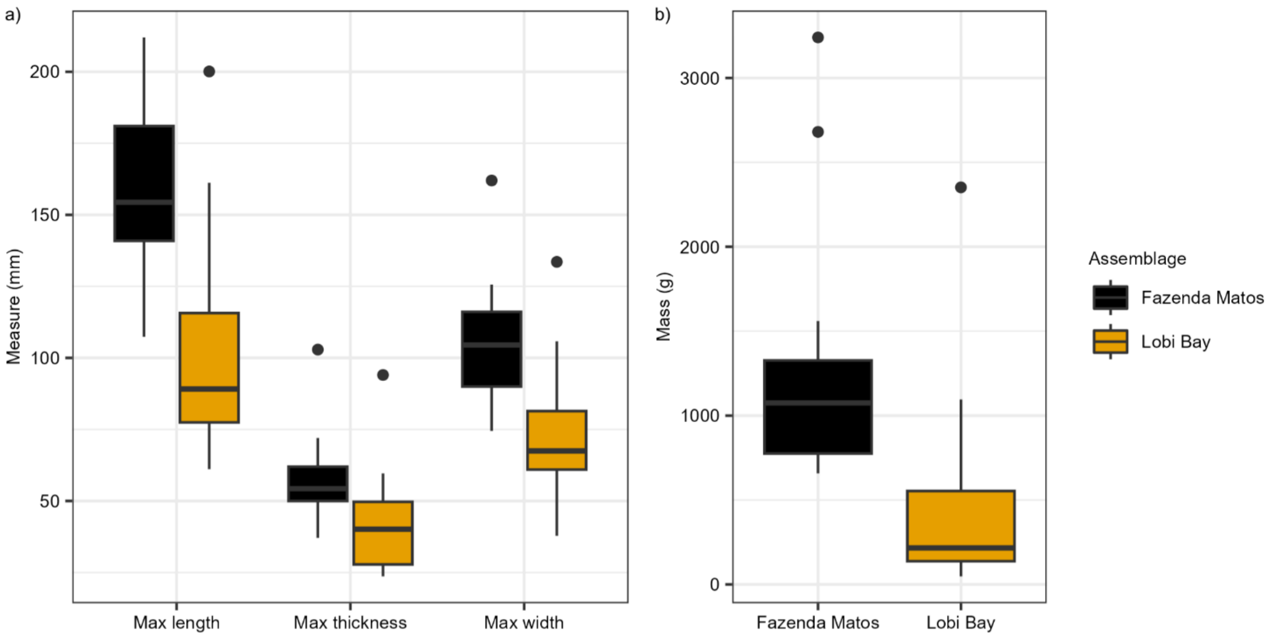

SOM Figure S7: Boxplot comparison of maximum dimensions and mass of flaked pieces from Fazenda Matos and Lobi Bay.

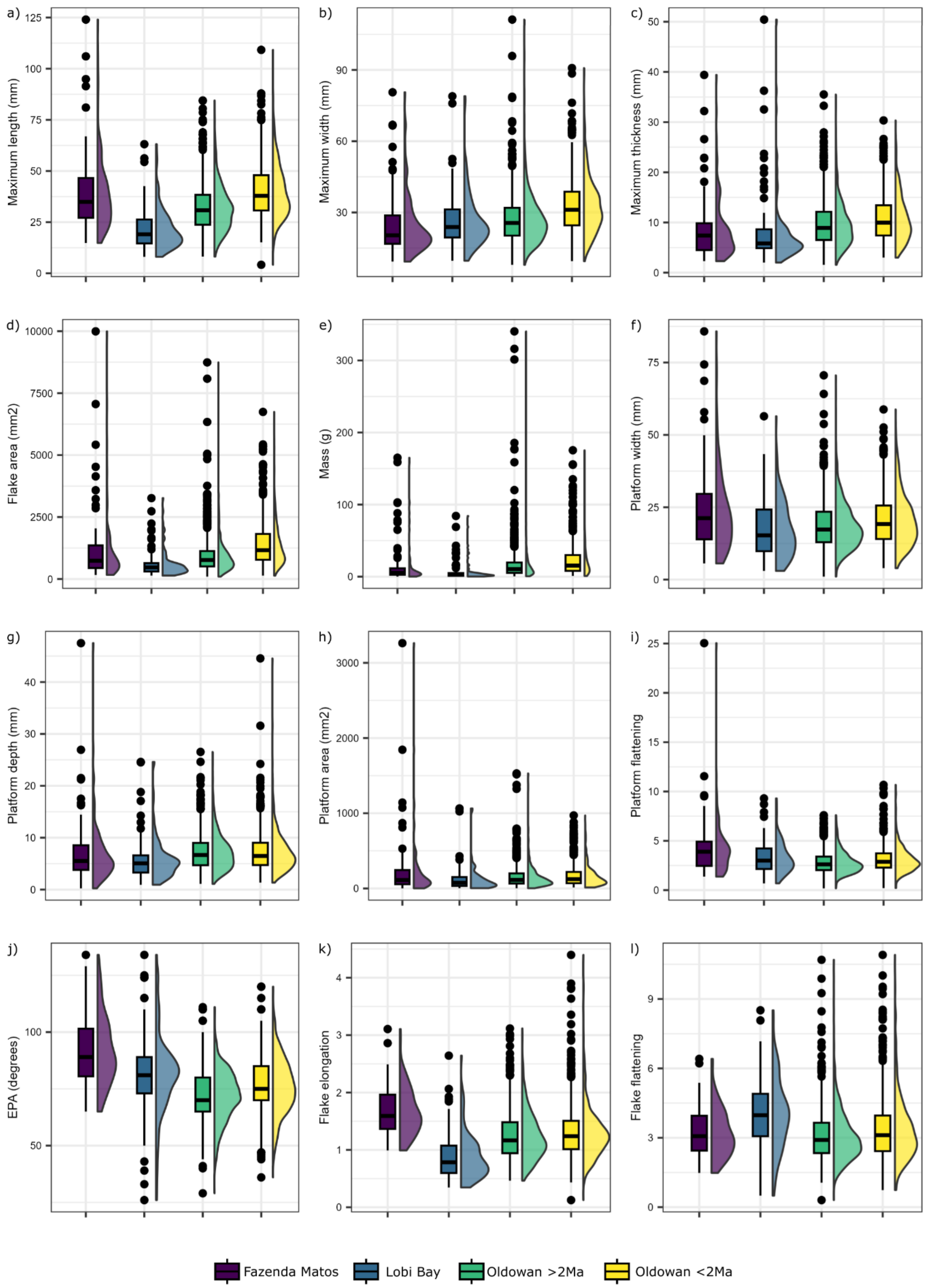

SOM Figure S8: Boxplot and violin plots comparison of complete flake length (a), width (b), thickness (c), flake area (d), mass (e), platform width (f), platform depth (g), platform area (h), platform flattening (i), external platform angle (EPA) (j), elongation (k), and flattening (l) between Fazenda Matos, Lobi Bay and Oldowan assemblages younger and older than 2Ma (data taken from (12).

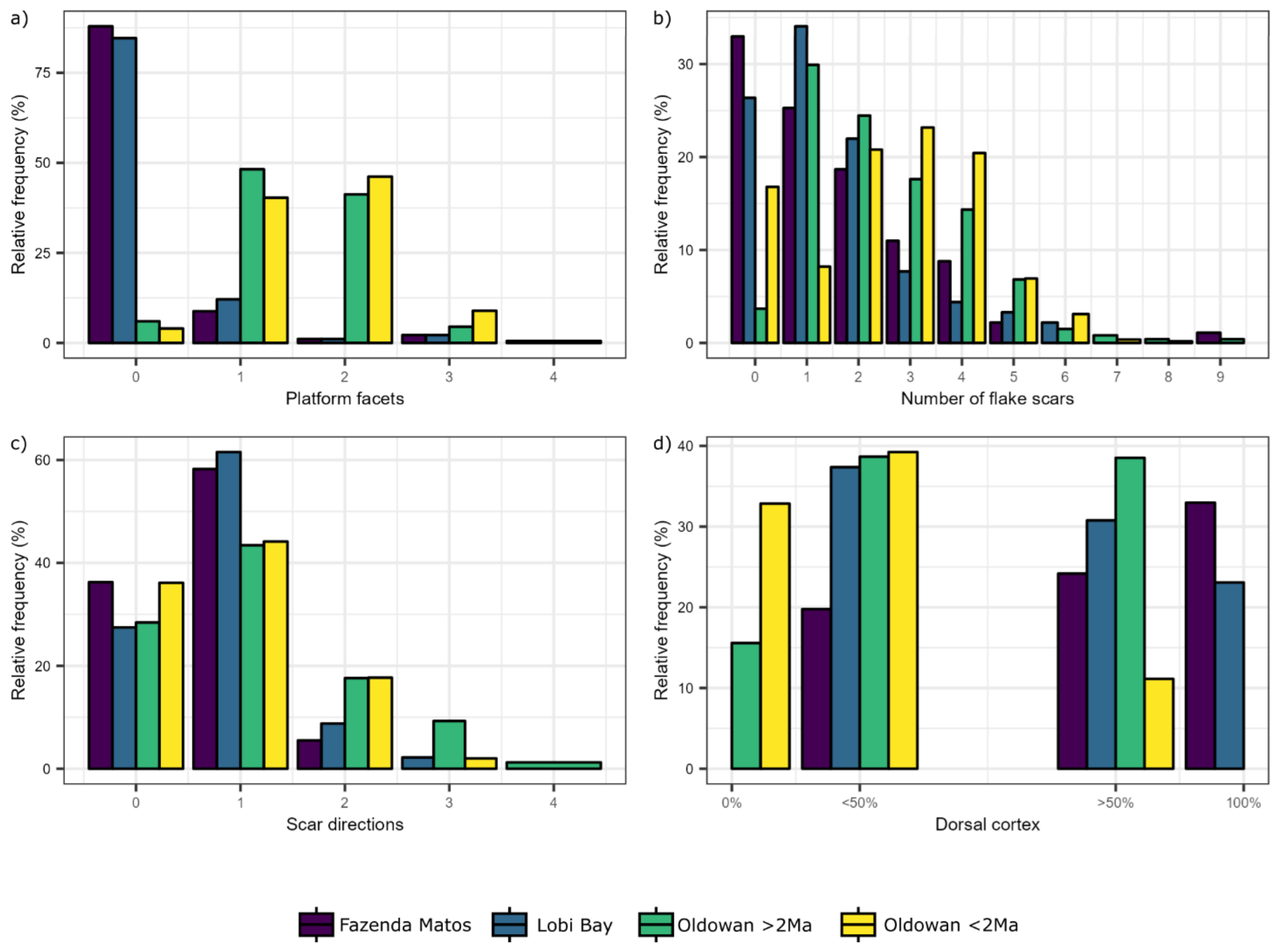

SOM Figure S9: Bar plot comparing relative frequencies of platform facets (a), number of dorsal flake scars (b), dorsal scar directions (c), dorsal cortex (d) on flakes between Fazenda Matos, Lobi Bay and Oldowan flake assemblages younger and older than 2Ma (data taken from (12).

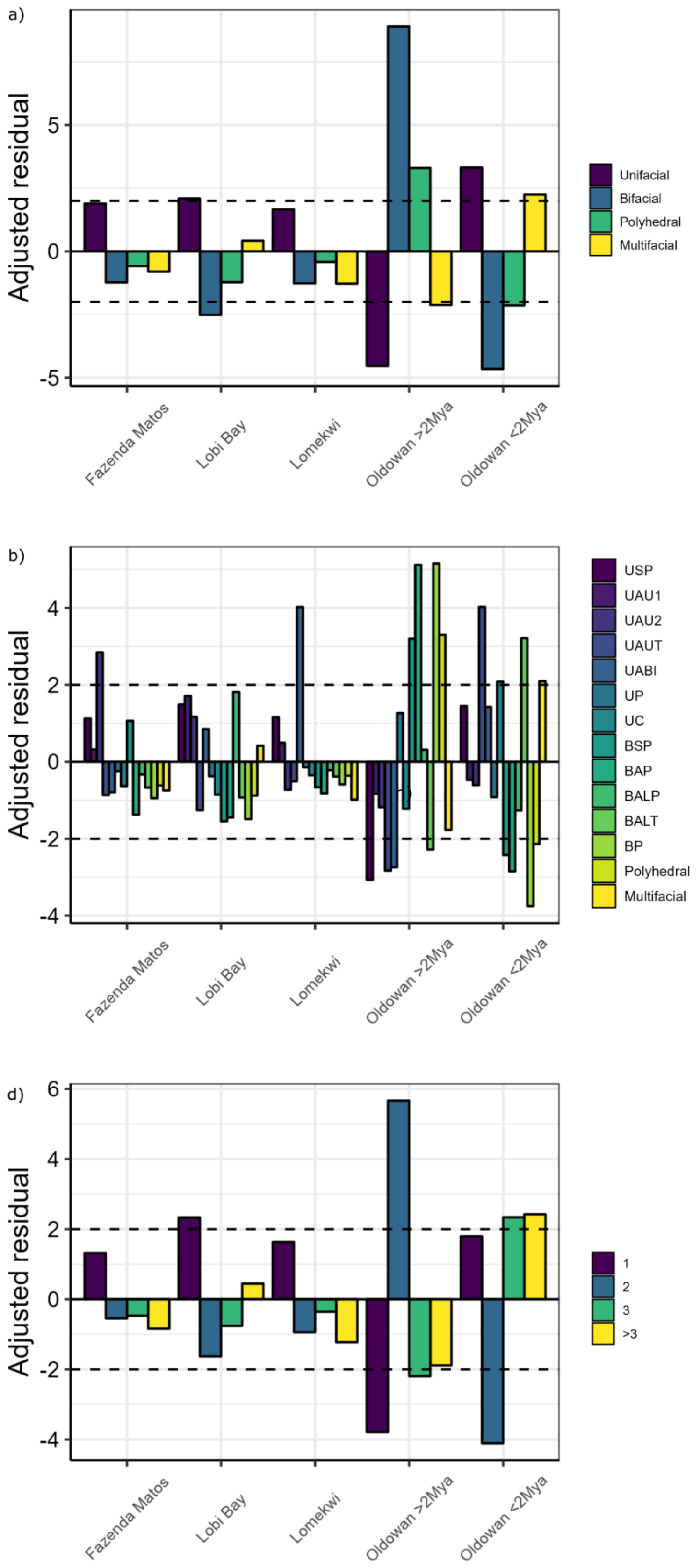

SOM Figure S10: Adjusted residual values for Chi Square test comparing primate and Plio-Pleistocene flaked pieces a) exploitation directions, b) exploitation patterns (USP: Unifacial simple partial exploitation;

UAU1: Unidirectional abrupt unifacial exploitation on one knapping surface; UAU2: Unidirectional abrupt unifacial exploitation on two independent knapping surfaces; UAUT: Unifacial abrupt unidirectional total exploitation; UABI: Unifacial abrupt bidirectional exploitation; BSP: Bifacial simple partial exploitation; BAP: Bifacial abrupt partial exploitation; BALP: Bifacial alternating partial exploitation; BALT: Bifacial alternating total exploitation; BP: Bifacial peripheral; UP: Unifacial peripheral exploitation; UC: Unifacial centripetal exploitation; Polyhedral: cores with three or more knapping surfaces, which become spherical; Multifacial: cores with three or more knapping surfaces with no organization of flaking, and c) number of flaked surfaces. A dashed line at y = 2 and y = -2 indicates significant over and under representation of each attribute.

Supplementary Tables

SOM Table S1: Absolute and relative frequencies and mass (g) of all technological categories and identified at Fazenda Matos separated by site.

Technological Categories	FM-001		FM-002		FM-003		FM-004		FM-005		FM-006		FM-007		FM-008		FM-009		FM-010		FM-011		Total Assemblage	
	n	%	n	%	n	%	n	%	n	%	n	%	n	%	n	%	n	%	n	%	n	%	n	%
Complete Hammerstone	0	0.0	0	0.0	0	0.0	0	0.0	0	0.0	0	0.0	0	0.0	2	3.1	0	0.0	0	0.0	0	0.0	2	0.6
Broken Hammerstone	0	0.0	0	0.0	2	5.6	1	4.5	3	8.3	3	0.0	0	0.0	0	0.0	0	0.0	0	0.0	0	0.0	9	2.5
Flaked Hammerstone	1	5.6	1	4.0	2	5.6	1	4.5	3	8.3	0	0.0	1	12.5	0	0.0	1	2.9	2	3.0	1	3.7	13	3.6
Complete Flake	6	33.3	11	44.0	10	27.8	5	22.7	13	36.1	2	0.0	5	62.5	12	18.8	5	14.3	18	27.3	5	18.5	92	25.8
Broken Flake	8	44.4	2	8.0	11	30.6	10	45.5	8	22.2	3	0.0	1	12.5	17	26.6	3	8.6	18	27.3	8	29.6	89	24.9
Hammerstone Flake	0	0.0	0	0.0	0	0.0	2	9.1	1	2.8	1	0.0	1	12.5	2	3.1	0	0.0	2	3.0	0	0.0	9	2.5
Retouched Piece	0	0.0	0	0.0	0	0.0	0	0.0	1	2.8	0	0.0	0	0.0	0	0.0	0	0.0	0	0.0	0	0.0	1	0.3
Angular Chunk	2	11.1	5	20.0	5	13.9	3	13.6	6	16.7	6	0.0	0	0.0	6	9.4	1	2.9	9	13.6	0	0.0	43	12.0
Small Debris	1	5.6	6	24.0	6	16.7	0	0.0	1	2.8	5	0.0	0	0.0	25	39.1	25	71.4	17	25.8	13	48.1	99	27.7
**Total**	**18**	**100**	**25**	**100**	**36**	**100**	**22**	**100**	**36**	**100**	**20**	**0.0**	**8**	**100**	**64**	**100**	**35**	**100**	**66**	**100**	**27**	**100**	**357**	**100**

Technological Categories	FM-001		FM-002		FM-003		FM-004		FM-005		FM-006		FM-007		FM-008		FM-009		FM-010		FM-011		Total Assemblage	
	g	%	g	%	g	%	g	%	g	%	g	%	g	%	g	%	g	%	g	%	g	%	n	%
Complete Hammerstone	0	0.0	0	0.0	0	0.0	0	0.0	0	0.0	0	0.0	0	0.0	1631.5	82.4	0	0.0	0	0.0	0	0.0	1631.5	6.5
Broken Hammerstone	0	0.0	0	0.0	415.5	13.8	324.2	9.0	1701	23.9	581.6	75.8	0	0.0	0	0.0	0	0.0	0	0.0	0	0.0	3022.3	12.0
Flaked Hammerstone	950.1	90.1	1320	75.8	2267.9	75.5	2680.4	74.4	4973.2	69.8	0	0.0	1327	88.3	0	0.0	882.8	80.5	1532.3	75.4	1180	94.2	17113.7	68.0
Complete Flake	27.2	2.6	231.8	13.3	65.2	2.2	202.6	5.6	152.2	2.1	7.7	1.0	165.9	11.0	181.7	9.2	187	17.0	192.1	9.4	38	3.0	1451.4	5.8
Broken Flake	15.1	1.4	37.3	2.1	72.1	2.4	320.5	8.9	61.8	0.9	3.1	0.4	5	0.3	57.4	2.9	16.1	1.5	152.7	7.5	32	2.6	773.1	3.1
Hammerstone Flake	0	0.0	0	0.0	0	0.0	56.5	1.6	22.4	0.3	15.7	2.0	5.5	0.4	8.2	0.4	0	0.0	24.9	1.2	0	0.0	133.2	0.5
Retouched Piece	0	0.0	0	0.0	0	0.0	0	0.0	68.5	1.0	0	0.0	0	0.0	0	0.0	0	0.0	0	0.0	0	0.0	68.5	0.3
Angular Chunk	60.6	5.7	149.2	8.6	178.7	5.9	19	0.5	147.3	2.1	157.9	20.6	0	0.0	88.9	4.5	5.5	0.5	121.6	6.0	0	0.0	928.7	3.7
Small Debris	1	0.1	3.1	0.2	6.2	0.2	0	0.0	1.7	0.0	1.7	0.2	0	0.0	11.6	0.6	5.9	0.5	9.7	0.5	2.9	0.2	43.8	0.2
**Total**	**1054.0**	**100**	**1741.40**	**100**	**3005.60**	**100**	**3603.20**	**100**	**7128.10**	**100**	**767.70**	**100.0**	**1503.40**	**100**	**1979.30**	**100**	**1097.30**	**100**	**2033.30**	**100**	**1252.90**	**100**	**25166.20**	**100**

SOM Table S2: Results of a Mann Whitney U test comparing the dimensions and mass of all complete and flaked hammerstones with natural stones from the study area.

	U	p
Maximum length (mm)	1520	0.877
Maximum width (mm)	1461	0.163
Maximum width (mm)	1310.5	0.553
Mass (g)	1559.5	0.055

SOM Table S3: Dimensions (mm and g) of all technological categories separated by site.

Technological Categories	FM-001				FM-002				FM-003				FM-004			
	Min	Max	Mean	SD	Min	Max	Mean	SD	Min	Max	Mean	SD	Min	Max	Mean	SD
**Complete Hammerstone**																
Maximum Length (mm)	-	-	-	-	-	-	-	-	-	-	-	-	-	-	-	-
Maximum Width (mm)	-	-	-	-	-	-	-	-	-	-	-	-	-	-	-	-
Maximum Thickness (mm)	-	-	-	-	-	-	-	-	-	-	-	-	-	-	-	-
Mass (g)	-	-	-	-	-	-	-	-	-	-	-	-	-	-	-	-
**Broken Hammerstone**																
Maximum Length (mm)	-	-	-	-	-	-	-	-	103.4	117.8	110.6	10.2	91.4	91.4	91.4	-
Maximum Width (mm)	-	-	-	-	-	-	-	-	51.1	65.3	58.2	10.0	70.4	70.4	70.4	-
Maximum Thickness (mm)	-	-	-	-	-	-	-	-	31.6	40.2	35.9	6.1	50.0	50.0	50.0	-
Mass (g)	-	-	-	-	-	-	-	-	171.2	244.3	207.8	51.7	324.2	324.2	324.2	-
**Flaked Hammerstone**																
Maximum Length (mm)	181.0	181.0	181.0	-	209.0	209.0	209.0	-	154.4	174.0	164.2	13.9	212.0	212.0	212.0	-
Maximum Width (mm)	90.0	90.0	90.0	-	112.0	112.0	112.0	-	113.0	117.4	115.2	3.1	162.0	162.0	162.0	-
Maximum Thickness (mm)	50.0	50.0	50.0	-	60.0	60.0	60.0	-	37.1	62.0	49.6	17.6	72.0	72.0	72.0	-
Mass (g)	950.1	950.1	950.1	-	1320.0	1320.0	1320.0	-	707.9	1560.0	1134.0	602.5	2680.4	2680.4	2680.4	-
**Complete Flake**																
Maximum Length (mm)	20.3	41.9	30.2	8.0	25.0	106.0	50.3	26.4	14.8	59.7	32.4	12.9	28.0	124.0	58.8	37.5
Maximum Width (mm)	11.5	28.5	20.7	6.9	13.4	66.6	27.0	15.5	14.3	36.2	23.2	8.1	18.7	80.6	38.4	24.5
Maximum Thickness (mm)	4.3	10.3	6.1	2.4	2.9	16.8	8.9	5.0	2.3	17.0	7.1	5.0	8.5	20.8	12.7	5.4
Mass (g)	1.6	9.5	4.5	3.1	1.2	103.0	21.1	35.0	0.6	23.0	6.5	7.5	5.3	158.9	40.5	66.5
**Broken Flake**																
Maximum Length (mm)	15.4	28.7	23.1	4.7	44.3	48.8	46.6	3.2	26.2	63.3	37.9	11.8	36.6	82.6	56.1	17.4
Maximum Width (mm)	11.7	23.2	16.1	3.6	24.7	41.9	33.3	12.1	13.0	42.8	21.7	9.6	18.7	73.3	34.9	19.5
Maximum Thickness (mm)	3.3	7.9	5.1	1.5	9.8	10.2	10.0	0.3	3.1	15.7	7.3	3.5	4.0	24.6	12.4	6.8
Mass (g)	1.1	3.2	1.9	0.8	12.5	24.8	18.7	8.7	1.3	22.7	6.6	7.0	2.3	178.4	32.1	53.1
**Hammerstone Flake**																
Maximum Length (mm)	-	-	-	-	-	-	-	-	-	-	-	-	43.2	68.0	55.6	17.5
Maximum Width (mm)	-	-	-	-	-	-	-	-	-	-	-	-	23.3	58.8	41.1	25.1
Maximum Thickness (mm)	-	-	-	-	-	-	-	-	-	-	-	-	6.4	10.6	8.5	3.0
Mass (g)	-	-	-	-	-	-	-	-	-	-	-	-	4.4	52.1	28.3	33.7
**Retouched Piece**																
Maximum Length (mm)	-	-	-	-	-	-	-	-	-	-	-	-	-	-	-	-
Maximum Width (mm)	-	-	-	-	-	-	-	-	-	-	-	-	-	-	-	-
Maximum Thickness (mm)	-	-	-	-	-	-	-	-	-	-	-	-	-	-	-	-
Mass (g)	-	-	-	-	-	-	-	-	-	-	-	-	-	-	-	-
**Angular Chunk**																
Maximum Length (mm)	24.1	66.9	45.5	30.2	19.1	71.1	51.1	21.8	37.3	68.2	55.9	13.9	28.0	37.8	34.1	5.3
Maximum Width (mm)	16.1	39.8	28.0	16.8	12.0	47.2	30.0	15.1	19.5	45.9	34.4	11.2	13.3	34.0	20.3	11.9
Maximum Thickness (mm)	9.7	20.2	14.9	7.4	6.9	26.1	16.5	8.9	6.2	39.4	23.4	12.9	7.3	11.6	9.6	2.2
Mass (g)	4.6	56.0	30.3	36.3	1.8	62.3	29.8	26.0	4.7	74.4	35.7	26.7	3.1	12.2	6.3	5.1
**Small Debris**																
Maximum Length (mm)	17.3	17.3	17.3	-	11.4	17.9	14.5	2.8	14.5	22.0	19.2	2.9	-	-	-	-
Maximum Width (mm)	10.3	10.3	10.3	-	6.2	17.5	10.5	4.7	10.1	15.6	12.1	1.9	-	-	-	-
Maximum Thickness (mm)	3.9	3.9	3.9	-	1.4	5.6	4.2	1.5	3.2	8.2	4.9	1.9	-	-	-	-
Mass (g)	1.0	1.0	1.0	-	0.1	1.5	0.5	0.7	0.1	2.7	1.0	1.0	-	-	-	-

Technological Categories	FM-005				FM-006				FM-007				FM-008			
	Min	Max	Mean	SD	Min	Max	Mean	SD	Min	Max	Mean	SD	Min	Max	Mean	SD
**Complete Hammerstone**																
Maximum Length (mm)	-	-	-	-	-	-	-	-	-	-	-	-	119.8	141.4	130.6	15.3
Maximum Width (mm)	-	-	-	-	-	-	-	-	-	-	-	-	70.2	76.5	73.4	4.5
Maximum Thickness (mm)	-	-	-	-	-	-	-	-	-	-	-	-	42.9	63.4	53.2	14.5
Mass (g)	-	-	-	-	-	-	-	-	-	-	-	-	582.8	1048.7	815.8	329.4
**Broken Hammerstone**																
Maximum Length (mm)	77.2	125.0	107.0	26.0	67.4	108.9	82.5	23.0	-	-	-	-	-	-	-	-
Maximum Width (mm)	57.9	93.7	77.9	18.3	49.9	90.7	64.4	22.8	-	-	-	-	-	-	-	-
Maximum Thickness (mm)	40.2	70.7	58.5	16.2	25.0	25.6	25.4	0.3	-	-	-	-	-	-	-	-
Mass (g)	155.0	780.3	567.0	356.9	143.6	245.0	193.9	50.7	-	-	-	-	-	-	-	-
**Flaked Hammerstone**																
Maximum Length (mm)	131.5	207.0	169.7	37.8	-	-	-	-	153.1	153.1	153.1	-	-	-	-	-
Maximum Width (mm)	81.9	125.6	97.7	24.2	-	-	-	-	116.1	116.1	116.1	-	-	-	-	-
Maximum Thickness (mm)	46.3	102.9	71.3	28.9	-	-	-	-	51.8	51.8	51.8	-	-	-	-	-
Mass (g)	658.0	3240.0	1657.7	1386.1	-	-	-	-	1327.0	1327.0	1327.0	-	-	-	-	-
**Complete Flake**																
Maximum Length (mm)	22.6	66.9	40.1	13.9	23.7	46.1	34.9	15.8	17.7	62.2	39.1	19.8	18.9	94.9	37.0	21.4
Maximum Width (mm)	17.5	48.3	26.0	9.5	14.4	23.8	19.1	6.6	9.3	57.6	34.4	19.8	9.4	47.7	21.9	10.9
Maximum Thickness (mm)	5.0	17.2	9.3	4.2	4.5	8.2	6.4	2.6	3.2	22.9	12.9	9.4	3.4	32.2	9.1	8.3
Mass (g)	3.0	39.5	11.7	11.9	1.1	6.6	3.9	3.9	0.1	88.1	33.2	40.5	0.9	102.3	15.1	29.2
**Broken Flake**																
Maximum Length (mm)	18.3	48.2	33.9	10.4	20.0	25.4	22.2	2.8	25.6	25.6	25.6	-	19.1	34.6	25.8	4.7
Maximum Width (mm)	13.8	42.2	23.5	9.6	13.3	14.6	14.1	0.7	19.9	19.9	19.9	-	10.7	22.6	16.7	3.9
Maximum Thickness (mm)	4.2	12.4	7.5	3.0	3.0	4.3	3.6	0.7	7.9	7.9	7.9	-	3.6	9.9	6.4	1.9
Mass (g)	1.5	20.6	7.7	8.1	0.9	1.2	1.0	0.2	5.0	5.0	5.0	-	1.2	9.1	3.4	2.0
**Hammerstone Flake**																
Maximum Length (mm)	66.8	66.8	66.8	-	41.0	41.0	41.0	-	35.9	35.9	35.9	-	29.1	29.8	29.5	0.5
Maximum Width (mm)	31.4	31.4	31.4	-	34.5	34.5	34.5	-	22.5	22.5	22.5	-	22.0	27.7	24.9	4.0
Maximum Thickness (mm)	10.6	10.6	10.6	-	12.5	12.5	12.5	-	5.3	5.3	5.3	-	6.3	7.0	6.7	0.5
Mass (g)	22.4	22.4	22.4	-	15.7	15.7	15.7	-	5.5	5.5	5.5	-	3.8	4.4	4.1	0.4
**Retouched Piece**																
Maximum Length (mm)	80.5	80.5	80.5	-	-	-	-	-	-	-	-	-	-	-	-	-
Maximum Width (mm)	59.7	59.7	59.7	-	-	-	-	-	-	-	-	-	-	-	-	-
Maximum Thickness (mm)	19.4	19.4	19.4	-	-	-	-	-	-	-	-	-	-	-	-	-
Mass (g)	68.5	68.5	68.5	-	-	-	-	-	-	-	-	-	-	-	-	-
**Angular Chunk**																
Maximum Length (mm)	22.1	91.6	47.4	25.1	19.7	71.2	42.5	22.8	13.3	64.5	32.4	18.9	-	-	-	-
Maximum Width (mm)	15.5	46.8	30.9	13.4	14.7	64.5	33.2	22.0	13.3	31.6	21.6	7.9	-	-	-	-
Maximum Thickness (mm)	8.2	27.1	16.3	7.9	6.8	16.0	12.0	4.3	5.9	20.2	13.3	7.1	-	-	-	-
Mass (g)	3.2	68.0	24.6	24.5	3.2	77.8	26.3	31.8	1.5	40.7	14.8	15.8	-	-	-	-
**Small Debris**																
Maximum Length (mm)	22.0	22.0	22.0	-	6.9	19.7	13.1	5.1	-	-	-	-	7.3	20.0	14.3	3.5
Maximum Width (mm)	14.3	14.3	14.3	-	4.1	13.6	7.8	3.8	-	-	-	-	5.1	14.2	9.7	2.6
Maximum Thickness (mm)	5.2	5.2	5.2	-	2.1	5.8	3.5	1.4	-	-	-	-	1.4	6.9	3.5	1.2
Mass (g)	1.7	1.7	1.7	-	0.1	1.3	0.3	0.5	-	-	-	-	0.1	1.9	0.5	0.5

Technological Categories	FM-009				FM-010				FM-011				Total Assemblage			
	Min	Max	Mean	SD	Min	Max	Mean	SD	Min	Max	Mean	SD	Min	Max	Mean	SD
**Complete Hammerstone**																
Maximum Length (mm)	-	-	-	-	-	-	-	-	-	-	-	-	119.8	141.4	130.6	15.3
Maximum Width (mm)	-	-	-	-	-	-	-	-	-	-	-	-	70.2	76.5	73.4	4.5
Maximum Thickness (mm)	-	-	-	-	-	-	-	-	-	-	-	-	42.9	63.4	53.2	14.5
Mass (g)	-	-	-	-	-	-	-	-	-	-	-	-				
**Broken Hammerstone**																
Maximum Length (mm)	-	-	-	-	-	-	-	-	-	-	-	-	67.4	125.0	97.9	21.9
Maximum Width (mm)	-	-	-	-	-	-	-	-	-	-	-	-	49.9	93.7	68.2	17.1
Maximum Thickness (mm)	-	-	-	-	-	-	-	-	-	-	-	-	25.0	70.7	41.5	17.1
Mass (g)	-	-	-	-	-	-	-	-	-	-	-	-	143.6	780.3	335.8	254.0
**Flaked Hammerstone**																
Maximum Length (mm)	107.4	107.4	107.4	-	138.4	140.9	139.7	1.8	146.2	146.2	146.2	-	107.4	212.0	163.5	32.4
Maximum Width (mm)	98.9	98.9	98.9	-	74.5	97.1	85.8	16.0	104.5	104.5	104.5	-	74.5	162.0	106.1	22.8
Maximum Thickness (mm)	60.7	60.7	60.7	-	48.5	50.2	49.4	1.2	54.3	54.3	54.3	-	37.1	102.9	58.5	16.1
Mass (g)	882.8	882.8	882.8	-	757.0	775.3	766.2	12.9	1180.0	1180.0	1180.0	-	658.0	3240.0	1316.4	786.8
**Complete Flake**																
Maximum Length (mm)	22.1	81.0	41.3	24.5	20.0	63.0	38.2	12.5	22.1	59.1	36.4	14.9	14.8	124.0	39.8	19.3
Maximum Width (mm)	14.9	66.9	31.1	21.1	11.2	46.3	21.5	9.3	14.5	33.7	20.7	8.3	9.3	80.6	25.0	13.0
Maximum Thickness (mm)	3.4	39.4	12.3	15.3	3.5	26.6	8.4	5.7	3.4	13.0	6.1	4.0	2.3	39.4	8.9	6.5
Mass (g)	1.2	165.0	37.4	71.5	1.1	75.3	10.7	17.2	1.1	26.0	7.6	10.6	0.1	165.0	15.8	30.4
**Broken Flake**																
Maximum Length (mm)	29.1	49.5	38.5	10.3	18.4	71.1	28.9	12.2	19.0	40.7	28.3	6.6	15.4	82.6	32.8	13.9
Maximum Width (mm)	13.1	27.7	20.0	7.3	9.8	57.1	19.9	11.6	12.3	24.5	18.1	4.3	9.8	73.3	21.1	11.2
Maximum Thickness (mm)	6.8	9.1	8.0	1.2	2.8	19.7	6.7	4.5	3.8	11.6	6.8	2.4	2.8	24.6	7.3	4.1
Mass (g)	1.0	8.6	5.4	3.9	1.1	74.8	8.5	18.5	1.4	7.6	4.0	2.1	0.9	178.4	8.7	21.1
**Hammerstone Flake**																
Maximum Length (mm)	-	-	-	-	25.3	49.5	37.4	17.1	-	-	-	-	25.3	68.0	43.2	15.7
Maximum Width (mm)	-	-	-	-	15.3	51.3	33.3	25.5	-	-	-	-	15.3	58.8	31.9	14.4
Maximum Thickness (mm)	-	-	-	-	4.9	13.4	9.2	6.0	-	-	-	-	4.9	13.4	8.6	3.2
Mass (g)	-	-	-	-	1.9	23.0	12.5	14.9	-	-	-	-	1.9	52.1	14.8	16.2
**Retouched Piece**																
Maximum Length (mm)	-	-	-	-	-	-	-	-	-	-	-	-	80.5	80.5	80.5	
Maximum Width (mm)	-	-	-	-	-	-	-	-	-	-	-	-	59.7	59.7	59.7	
Maximum Thickness (mm)	-	-	-	-	-	-	-	-	-	-	-	-	19.4	19.4	19.4	
Mass (g)	-	-	-	-	-	-	-	-	-	-	-	-	68.5	68.5	68.5	
**Angular Chunk**																
Maximum Length (mm)	26.1	26.1	26.1	-	26.7	73.2	39.0	15.0	-	-	-	-	13.3	91.6	42.7	19.3
Maximum Width (mm)	19.9	19.9	19.9	-	12.3	45.8	22.5	10.2	-	-	-	-	12.0	64.5	27.3	13.5
Maximum Thickness (mm)	11.2	11.2	11.2	-	7.1	18.5	11.7	4.0	-	-	-	-	5.9	39.4	14.5	7.7
Mass (g)	5.5	5.5	5.5	-	3.3	60.2	13.5	18.2	-	-	-	-	1.5	77.8	21.6	23.1
**Small Debris**																
Maximum Length (mm)	7.8	20.7	12.6	3.4	10.6	20.6	16.4	2.8	8.0	16.4	12.4	2.5	6.9	22.0	14.3	3.7
Maximum Width (mm)	4.3	14.7	8.8	2.9	7.0	16.3	11.0	2.7	5.8	13.0	8.1	1.9	4.1	17.5	9.6	3.0
Maximum Thickness (mm)	1.1	6.8	2.8	1.3	1.6	4.6	2.8	0.9	1.5	4.3	2.4	0.8	1.1	8.2	3.2	1.4
Mass (g)	0.1	2.2	0.2	0.4	0.1	1.3	0.6	0.4	0.1	0.7	0.2	0.2	0.1	2.7	0.4	0.5

SOM Table S4: Absolute and relative frequencies of technological attributes recorded on flaked pieces from Fazenda Matos.

Attribute	n	%
**Blank morphology**		
Tabular	12	92.3
Plano-convex	1	7.7
**Num of platforms**		
1	3	23.1
2	10	76.9
**Platform battering**		
Present	13	100.0
Absent	0	0.0
**Edge Battering**		
Present	11	84.6
Absent	2	15.4
**Cortex**		
0%	0	0.0
<50%	0	0.0
>50%	13	100.0
**Exploitation**		
Unifacial	10	76.9
Bifacial	2	15.4
Multifacial	1	7.7
**Exploitation pattern**		
USP	4	30.8
UAU1	2	15.4
UAU2	4	30.8
BSP	2	15.4
Multifacial	1	7.7

SOM Table S5: Quantitative technological attributes of all flaked pieces from Fazenda Matos.

Attribute		Value
**Num flake scars (>10mm)**		
	Minimum	2
	Maximum	22
	Mean	9.2
	SD	5.7
**Flake scar length (mm)**		
	Minimum	9.58
	Maximum	89.31
	Mean	27.91
	SD	16.71
**Flake scar width (mm)**		
	Minimum	9.65
	Maximum	89.54
	Mean	39.25
	SD	18.62
**Flake scar elongation**		
	Minimum	0.30
	Maximum	2.13
	Mean	0.75
	SD	0.34

SOM Table S6: Absolute and relative frequencies of technological attributes for all complete flakes at Fazenda Matos.

Attribute	n	%
**Impact point location**		
Centred	55	59.8
De-centred	37	40.2
**Platform cortex**		
100%	81	88.0
>50%	0	0.0
<50%	1	1.1
0%	10	10.9
**Platfrom morphology**		
Rectilinear	75	81.5
Lineal	16	17.4
Convex	1	1.1
**Platform facets**		
Non-faceted	81	88.0
Uni-faceted	8	8.7
Bi-faceted	1	1.1
Multi-faceted	2	2.2
**Platform shape**		
Rectilinear	81	88.0
Convex	9	9.8
Uni-angular	2	2.2
**Flake termination**		
Feather	62	67.4
Hinge	18	19.6
Step	6	6.5
Plunging	6	6.5
**Step scars**		
Present	32	34.8
Absent	60	65.2
**Step scar stacks**		
Present	2	2.2
Absent	90	97.8
**Bulb morphology**		
Marked	14	15.2
Disffused	53	57.6
Fractured	2	2.2
Indeterminante	23	25.0
**Dorsal cortex**		
100%	30	32.6
>50%	22	23.9
<50%	18	19.6
0%	22	23.9
**Number of dorsal scars**		
0	30	32.6
1-3	51	55.4
4-5	10	10.9
>5	1	1.1
**Toth flake category**		
I	26	28.3
II	36	39.1
III	19	20.7
IV	4	4.3
V	4	4.3
VI	3	3.3

SOM Table S7: Adjusted residuals associated with the Chi Square comparison between frequency of technological categories at Fazenda Matos and Lobi Bay including all raw materials and only high-quality raw materials.

	**All Raw Material**		**Only high-quality RM**	
	Fazenda Matos	Lobi Bay	Fazenda Matos	Lobi Bay
**Technological Category**	**Adj residual**		**Adj residual**	
Angular debris	**-8.5**	**4.8**	-1.2	1.0
Angular debris (Percussion)	**-4.8**	**2.7**	-0.4	0.4
Flake	**6.4**	**-3.6**	0.6	-0.5
Flaked Piece	1.2	-0.7	-0.9	0.8
Fragmented flake	**7.2**	**-4.1**	1.4	-1.3
Hammerstone	**-3.6**	**2.1**	**-3.0**	**2.7**
Hammerstone flake	0.1	0.0	-1.8	1.6
Hammerstone fragment	-1.3	0.7	0.4	-0.3
Retouched Piece	1.5	-0.9	0.8	-0.7
Small debris <20mm	**7.3**	**-4.2**	1.2	-1.0

SOM Table S8: Results of a Mann Whitney U comparison of maximum dimensions, mass, elongation, flattening and number of extraction values for all flaked pieces from Fazenda Matos and Lobi Bay. Significant values are highlighted in bold.

	**Mann Whitney U**	
	**U**	***p***
Maximum Length (mm)	308	**<0.001**
Maximum Width (mm)	300	**<0.001**
Maximum Thickness (mm)	283	**<0.001**
Mass (g)	315	**<0.001**
Elongation	232	0.063
Flattening	234	0.054
Number of extractions	207	0.262

SOM Table S9 Results of a Chi Square Test comparison of technological attributes for all flaked pieces from Fazenda Matos and Lobi Bay. Significant values are highlighted in bold.

		**Mann Whitney U**	
	χ^2	**df**	***p***
Flaking Pattern	9.500	8	0.302
Exploitation	1.267	2	0.531
Cortical coverage	<0.001	1	1.000
Step scars	5.687	1	**0.017**

SOM Table S10: Absolute and relative frequencies of technological` attributes of flaked pieces from Fazenda Matos and Lobi Bay (1).

**Attribute**	**Fazenda Matos**		**Lobi Bay**	
**Flaking pattern**	***n***	**%**	***n***	**%**
BALP	0	0.00	1	3.85
BAP	0	0.00	1	3.85
BSP	2	15.38	0	0.00
Multifacial	1	7.69	5	19.23
UABI	0	0.00	2	7.69
UAU1	2	15.38	6	23.08
UAU2	4	30.77	4	15.38
USP	4	30.77	5	19.23
USP2	0	0.00	2	7.69
**Exploitation**				
Bifacial	2	15.38	2	7.69
Multifacial	1	7.69	5	19.23
Unifacial	10	76.92	19	73.08
**Percussive damage**				
Yes	13	100.00	26	100.00
No	0	0.00	0	0.00
**Cortex**				
100%				
>50%	13	100.00	25	96.15
<50%	0	0.00	1	3.85
0%				
**Number of extractions**				
1-3	8	6.72	13	6.88
4-6	11	9.24	41	21.69
7-9	26	21.85	39	20.63
10-13	23	19.33	42	22.22
14-16	29	24.37	14	7.41
17-20	0	0.00	19	10.05
>20	22	18.49	21	11.11
**Step scars**				
No	2	15.38	16	61.54
Yes	11	84.62	10	38.46

SOM Table S11: Results of a Kruskal Wallis test comparing quantitative flake attributes between Fazenda Matos, Lobi Bay and Oldowan assemblages alongside the results of Bonferroni adjusted Dunns Post Hoc tests (significant results are highlighted in bold, data for Lobi Bay taken from (1), data for Oldowan assemblages taken from (12)).

	Kruskall Wallis			Dunns Post Hoc Test					
**Attribute**	X2	df	p	FM-LB	FM-Old<2Ma	LB-Old<2Ma	FM-Old>2Ma	LB-Old>2Ma	Old >2Ma -
Length (mm)	235.28	3	**<0.001**	**<0.001**	0.402	**<0.001**	**0.001**	**<0.001**	**<0.001**
Width (mm)	120.49	3	**<0.001**	0.696	**<0.001**	**<0.001**	**0.006**	1.000	**<0.001**
Thickness (mm)	77.663	3	**<0.001**	0.768	**<0.001**	**<0.001**	**0.004**	**<0.001**	**<0.001**
Flake Area (mm2)	77.663	3	**<0.001**	0.768	**<0.001**	**<0.001**	**0.004**	**<0.001**	**<0.001**
Flake Volume (mm3)	153.76	3	**<0.001**	**0.007**	**<0.001**	**<0.001**	0.806	**<0.001**	**<0.001**
Mass (g)	187.87	3	**<0.001**	**0.004**	**<0.001**	**<0.001**	**<0.001**	**<0.001**	**<0.001**
Platform width (mm)	26.513	3	**<0.001**	**0.002**	1.000	**0.002**	0.016	0.418	**0.002**
Platform depth (mm)	24.958	3	**<0.001**	0.658	0.151	**<0.001**	0.120	**<0.001**	1.000
Platform area (mm2)	20.018	3	**<0.001**	**0.028**	1.000	**<0.001**	1.000	**0.003**	0.260
Platform flattening	50.326	3	**<0.001**	0.058	**<0.001**	1.000	**<0.001**	**0.029**	**<0.001**
EPA (degrees)	159.63	3	**<0.001**	**<0.001**	**<0.001**	**0.012**	**<0.001**	**<0.001**	**<0.001**
Flake elongation	155.07	3	**<0.001**	**<0.001**	**<0.001**	**<0.001**	**<0.001**	**<0.001**	**0.043**
Flake flattening	42.567	3	**<0.001**	**0.002**	1.000	**<0.001**	1.000	**<0.001**	**0.003**

SOM Table S12: Chi Square Test results for all technological attributes compared between Fazenda Matos and Lobi Bay on complete flakes.

	**Chi Square Test**		
	x^2	**df**	p
Impact point location	0.000	1	1.000
Platform cortex	3.985	3	0.263
Platform type	10.668	4	**0.031**
Platform faceting	0.570	3	0.903
Platform shape	7.811	3	0.050
Ventral surface morphology	4.882	4	0.300
Bulb of percussion	3.409	3	0.333
Dorsal cortex	13.760	3	**0.003**
Flake categories	14.191	5	**0.014**
Flake termination	4.480	3	0.214
Step scars	7.096	1	**0.008**

SOM Table S13: Adjusted residual values of Chi Square Test results for technological attribute comparison between complete flakes from Fazenda Matos and Lobi Bay

	Fazenda Matos	Lobi Bay
**Technological Category**	**Adj residual**	
**Impact point location**		
Centred	-0.040	0.041
De-centred	0.050	-0.050
**Platfrom cortex**		
100	0.176	-0.177
>50%	-0.709	0.713
<50%	-1.161	1.167
0	0.316	-0.318
**Platform type**		
Rectilinear	-0.048	0.048
Lineal	1.133	-1.139
Punctiform	-1.737	1.746
Crushed	-0.709	0.713
**Platform faceting**		
Non-faceted	0.176	-0.177
Uni-faceted	-0.502	0.505
Bi-faceted	-0.005	0.005
Multi-faceted	-0.008	0.008
**Platform shape**		
Rectilinear	0.826	-0.830
Concave	-1.003	1.008
Convex	-1.241	1.248
Uni-angular	-0.810	0.814
**Ventral surface morphology**		
Rectilinear	-0.314	0.315
Concave	0.980	-0.986
Convex	-0.458	0.461
Uni-angular	-0.709	0.713
Irregular	-0.810	0.814
**Bulb of percussion**		
Marked	-0.963	0.969
Diffused	0.385	-0.387
Fractured	-0.585	0.588
indeterminate	0.526	-0.529
**Dorsal cortex**		
100	0.861	-0.866
>50%	-0.626	0.629
<50%	-1.592	1.601
0	1.781	-1.791
**Flake categories**		
I	0.710	-0.714
II	-1.239	1.246
III	1.814	-1.824
IV	0.566	-0.569
V	-1.145	1.152
VI	0.307	-0.308
**Flake termination**		
Feather	-0.415	0.417
Hinge	1.363	-1.371
Step	-0.391	0.393
Plunging	-0.209	0.211
**Step scars**		
Present	1.722	-1.732
Absent	-1.012	1.018

SOM Table S14: Technological attributes of Plio-Pleistocene archaeological sites and primate flaked assemblages. All archaeological data is derived from (13).

Site	Industry	% cores in assemblage	% angular fragments	Mean core size (mm)	Ratio mean flake size to mean core size	Mean flake scar count	Ratio of flake scar count to log10 (mean core size)	Mean flake scar maximum dimension (mm)	Mean flake thickness (mm)	% percussive pieces
EFHR	Acheulean	22.6	39.5	94.9	0.6	10.2	5.2	58	16	0.9
FxJj63	Acheulean	6.7	37.9	108.9	0.6	7	3.4	60	10.2	0.3
FxJj37	Acheulean	8.9	26.7	84.6	0.8	7.1	3.7	66	15.2	4.4
Peninj_ST	Acheulean	7.1	24.2	62.2	0.5	9.4	5.2	40.5	12.3	3.1
FxJj20Main	Oldowan <2Ma	2	63.7	50.5	0.5	7.1	4.2	26	6.5	0.1
FxJj18IHS	Oldowan <2Ma	3.5	55.1	52.9	0.7	9.1	5.3	37	8.3	0.2
DK	Oldowan <2Ma	16.1	40.1	67.9	0.6	7.2	3.9	40.2	11.9	3
FLKZinj	Oldowan <2Ma	3.4	56.5	76.4	0.5	5.8	3.1	36.8	11.5	1.2
FxJj1	Oldowan <2Ma	13.8	46.7	54.4	0.7	5.4	3.1	39	11.3	0.6
FxJj10	Oldowan <2Ma	6.3	48.4	58.6	0.7	5.3	3	40.4	11.9	0.2
Omo57	Oldowan <2Ma	1.4	35.9	37.4	0.7	3.2	2	24.7	7.7	0.6
Omo123	Oldowan <2Ma	1.2	41.4	30.5	0.6	3.2	2.2	17.7	6.4	0.2
KJS	Oldowan <2Ma	11.2	50.4	56.3	0.6	5.4	3.1	32.6	9.9	0.1
EO	Oldowan >2Ma	10.3	18.8	70.9	10.4	6.3	3.4	30.4	12.5	2.8
Fejej	Oldowan <2Ma	3.5	35.9	58.3	0.6	5.1	2.9	36.9	10.7	7
AL894	Oldowan >2Ma	0.8	16.9	75	0.5	6.9	3.7	35.9	10.5	0
OGS7	Oldowan >2Ma	3.8	44.9	44.1	0.9	5.4	3.3	39.1	11.8	1.1
EG10	Oldowan >2Ma	5.6	57.2	83.3	0.4	7.3	3.8	36.1	13.6	0
EG12	Oldowan >2Ma	1.5	41.3	74.5	0.5	5.4	2.9	34.6	12.8	0
BD1	Oldowan >2Ma	13	23.4	58	0.6	2.9	1.6	32.4	9.4	1.3
NYA	Oldowan >2Ma	20.6	51.2	72.7	0.6	5.1	2.7	43.1	13.9	7
Sare	Oldowan <2Ma	0.5	93	47.9	0.7	5.2	3.1	34.6	8	0
HWK_EE	Oldowan <2Ma	5.5	71.5	70.2	0.4	4.3	2.3	31	11.8	4.2
FM	Fazenda Matos Capuchin	3.6	19.2	163.5	0.2	9.2	4.2	39.8	8.9	6.7

LB	Lobi Bay Macaque	2.3	32	99.7	0.3	7	3.5	32.6	8.2	8.1
Lom3	Lomekwi	55.7	20	147.8	0.7	4.7	2.2	120	43.9	9.4
SCNP	Capuchin SoS	21	26.12	71.73	0.54	1.43	1.61	44.34	13.2	33.3

SOM Tabe S15: Results of a pairwise Brown Forsythe test for all quantitative attributes of primate and Oldowan flake assemblages (significant results are highlighted in bold).

		**Pairwise Brown Forsythe test**		
**Attribute**	**Comparison**	df	F	p
Length				
	Oldowan >2Mya vs Lobi Bay	1	3.969	**0.047**
	Oldowan >2Mya vs Fazenda Matos	1	19.007	**<0.001**
	Oldowan <2Mya vs Lobi Bay	1	11.559	**0.001**
	Oldowan <2Mya vs Fazenda Matos	1	5.936	**0.015**
Width				
	Oldowan >2Mya vs Lobi Bay	1	0.217	0.641
	Oldowan >2Mya vs Fazenda Matos	1	0.892	0.345
	Oldowan <2Mya vs Lobi Bay	1	1.351	0.245
	Oldowan <2Mya vs Fazenda Matos	1	0.436	0.509
Thickness				
	Oldowan >2Mya vs Lobi Bay	1	0.881	0.348
	Oldowan >2Mya vs Fazenda Matos	1	5.139	**0.024**
	Oldowan <2Mya vs Lobi Bay	1	0.081	0.776
	Oldowan <2Mya vs Fazenda Matos	1	2.394	0.122
Flake area				
	Oldowan >2Mya vs Lobi Bay	1	2.576	0.109
	Oldowan >2Mya vs Fazenda Matos	1	12.039	**0.001**
	Oldowan <2Mya vs Lobi Bay	1	16.517	**<0.001**
	Oldowan <2Mya vs Fazenda Matos	1	0.958	0.328
Flake volume				
	Oldowan >2Mya vs Lobi Bay	1	1.300	0.255
	Oldowan >2Mya vs Fazenda Matos	1	9.005	**0.003**
	Oldowan <2Mya vs Lobi Bay	1	10.171	**0.001**
	Oldowan <2Mya vs Fazenda Matos	1	3.244	0.072
Mass				
	Oldowan >2Mya vs Lobi Bay	1	5.353	**0.021**
	Oldowan >2Mya vs Fazenda Matos	1	0.188	0.665
	Oldowan <2Mya vs Lobi Bay	1	18.773	**<0.001**
	Oldowan <2Mya vs Fazenda Matos	1	0.659	0.417
Platform width				
	Oldowan >2Mya vs Lobi Bay	1	5.808	**0.016**
	Oldowan >2Mya vs Fazenda Matos	1	38.656	**<0.001**
	Oldowan <2Mya vs Lobi Bay	1	0.637	0.425
	Oldowan <2Mya vs Fazenda Matos	1	19.952	**<0.001**
Platform depth				
	Oldowan >2Mya vs Lobi Bay	1	0.003	0.954
	Oldowan >2Mya vs Fazenda Matos	1	8.761	**0.003**
	Oldowan <2Mya vs Lobi Bay	1	0.036	0.850
	Oldowan <2Mya vs Fazenda Matos	1	4.851	**0.028**
Platform area				
	Oldowan >2Mya vs Lobi Bay	1	0.171	0.679
	Oldowan >2Mya vs Fazenda Matos	1	18.797	**<0.001**
	Oldowan <2Mya vs Lobi Bay	1	0.012	0.912
	Oldowan <2Mya vs Fazenda Matos	1	14.342	**<0.001**
Plaform flattening				
	Oldowan >2Mya vs Lobi Bay	1	14.462	**<0.001**
	Oldowan >2Mya vs Fazenda Matos	1	43.608	**<0.001**
	Oldowan <2Mya vs Lobi Bay	1	4.743	**0.030**
	Oldowan <2Mya vs Fazenda Matos	1	22.872	**<0.001**
EPA				
	Oldowan >2Mya vs Lobi Bay	1	21.503	**<0.001**
	Oldowan >2Mya vs Fazenda Matos	1	15.538	**<0.001**
	Oldowan <2Mya vs Lobi Bay	1	9.159	**0.003**
	Oldowan <2Mya vs Fazenda Matos	1	5.447	**0.020**
Flake elongation				
	Oldowan >2Mya vs Lobi Bay	1	0.638	0.425
	Oldowan >2Mya vs Fazenda Matos	1	0.010	0.920
	Oldowan <2Mya vs Lobi Bay	1	0.792	0.374
	Oldowan <2Mya vs Fazenda Matos	1	0.045	0.832
Flake flattening				
	Oldowan >2Mya vs Lobi Bay	1	18.718	**<0.001**
	Oldowan >2Mya vs Fazenda Matos	1	0.012	0.912
	Oldowan <2Mya vs Lobi Bay	1	2.111	0.147
	Oldowan <2Mya vs Fazenda Matos	1	3.523	0.061

SOM Table S16: Results of a pairwise Dunn's test for all quantitative attributes of primate and Oldowan flake assemblages (significant results are highlighted in bold).

		Pairwise Dunn's Test		
**Attribute**	**Comparison**	Z	P.unadj	P.adj
Length	Oldowan >2Mya vs Lobi Bay	-7.620	**<0.001**	**<0.001**
	Oldowan >2Mya vs Fazenda Matos	3.812	**<0.001**	**0.001**
	Oldowan <2Mya vs Lobi Bay	-13.058	**<0.001**	**<0.001**
	Oldowan <2Mya vs Fazenda Matos	-1.832	0.067	0.402
Width	Oldowan >2Mya vs Lobi Bay	-1.184	0.236	1.000
	Oldowan >2Mya vs Fazenda Matos	-3.281	**0.001**	**0.006**
	Oldowan <2Mya vs Lobi Bay	-5.710	**<0.001**	**<0.001**
	Oldowan <2Mya vs Fazenda Matos	-7.769	**<0.001**	**<0.001**
Thickness	Oldowan >2Mya vs Lobi Bay	-5.452	**<0.001**	**<0.001**
	Oldowan >2Mya vs Fazenda Matos	-3.423	**0.001**	**0.004**
	Oldowan <2Mya vs Lobi Bay	-7.500	**<0.001**	**<0.001**
	Oldowan <2Mya vs Fazenda Matos	-5.507	**<0.001**	**<0.001**
Flake area	Oldowan >2Mya vs Lobi Bay	-5.652	**<0.001**	**<0.001**
	Oldowan >2Mya vs Fazenda Matos	0.208	0.835	1.000
	Oldowan <2Mya vs Lobi Bay	-11.135	**<0.001**	**<0.001**
	Oldowan <2Mya vs Fazenda Matos	-5.381	**<0.001**	**<0.001**
Flake volume	Oldowan >2Mya vs Lobi Bay	-5.804	**<0.001**	**<0.001**
	Oldowan >2Mya vs Fazenda Matos	-1.497	0.134	0.806
	Oldowan <2Mya vs Lobi Bay	-10.199	**<0.001**	**<0.001**
	Oldowan <2Mya vs Fazenda Matos	-5.970	**<0.001**	**<0.001**
Mass	Oldowan >2Mya vs Lobi Bay	-8.793	**<0.001**	**<0.001**
	Oldowan >2Mya vs Fazenda Matos	-4.280	**<0.001**	**<0.001**
	Oldowan <2Mya vs Lobi Bay	-12.116	**<0.001**	**<0.001**
	Oldowan <2Mya vs Fazenda Matos	-7.685	**<0.001**	**<0.001**
Platform width	Oldowan >2Mya vs Lobi Bay	-1.814	0.070	0.418
	Oldowan >2Mya vs Fazenda Matos	3.007	**0.003**	**0.016**
	Oldowan <2Mya vs Lobi Bay	-3.606	**<0.001**	**0.002**
	Oldowan <2Mya vs Fazenda Matos	1.129	0.259	1.000
Platform depth	Oldowan >2Mya vs Lobi Bay	-4.459	**<0.001**	**<0.001**
	Oldowan >2Mya vs Fazenda Matos	-2.326	**0.020**	0.120
	Oldowan <2Mya vs Lobi Bay	-4.333	**<0.001**	**<0.001**
	Oldowan <2Mya vs Fazenda Matos	-2.238	**0.025**	0.151
Platform area	Oldowan >2Mya vs Lobi Bay	-3.454	**0.001**	**0.003**
	Oldowan >2Mya vs Fazenda Matos	0.314	0.754	1.000
	Oldowan <2Mya vs Lobi Bay	-4.400	**<0.001**	**<0.001**
	Oldowan <2Mya vs Fazenda Matos	-0.700	0.484	1.000
	Comparison			
Plaform flattening		2.816	**0.005**	**0.029**
	Oldowan >2Mya vs Fazenda Matos	6.266	**<0.001**	**<0.001**
	Oldowan <2Mya vs Lobi Bay	0.660	0.509	1.000
	Oldowan <2Mya vs Fazenda Matos	4.048	**<0.001**	**<0.001**
EPA	Oldowan >2Mya vs Lobi Bay	6.038	**<0.001**	**<0.001**
	Oldowan >2Mya vs Fazenda Matos	11.446	**<0.001**	**<0.001**
	Oldowan <2Mya vs Lobi Bay	3.094	**0.002**	**0.012**
	Oldowan <2Mya vs Fazenda Matos	8.404	**<0.001**	**<0.001**
Flake elongation	Oldowan >2Mya vs Lobi Bay	-7.546	**<0.001**	**<0.001**
	Oldowan >2Mya vs Fazenda Matos	8.670	**<0.001**	**<0.001**
	Oldowan <2Mya vs Lobi Bay	-8.752	**<0.001**	**<0.001**
	Oldowan <2Mya vs Fazenda Matos	7.172	**<0.001**	**<0.001**
Flake flattening	Oldowan >2Mya vs Lobi Bay	6.121	**<0.001**	**<0.001**
	Oldowan >2Mya vs Fazenda Matos	1.344	0.179	1.000
	Oldowan <2Mya vs Lobi Bay	4.252	**<0.001**	**<0.001**
	Oldowan <2Mya vs Fazenda Matos	-0.439	0.661	1.000

SOM Table S17: Results of a Chi Square Test of a comparison of technological attributes on complete flake attributes between Fazenda Matos, Lobi Bay and Oldowan assemblages (significant results are highlighted in bold).

	Chi Square test		
**Attribute**	X2	df	p
Platform facets	836.76	12	**<0.001**
Num flake scars	233.34	27	**<0.001**
Scar directions	74.051	12	**<0.001**
Dorsal cortex	204.38	9	**<0.001**

SOM Table S18: Adjusted residual values for the Chi Square Test comparison of technological attributes on complete flake attributes between Fazenda Matos, Lobi Bay and Oldowan assemblages (significant results are highlighted in bold).

Attribute		Adjusted residuals			
		Fazenda Matos	Lobi Bay	Oldowan >2Ma	Oldowan <2Ma
Platform facets					
	0	**19.91**	**22.69**	**-11.52**	**-8.78**
	1	**-4.92**	**-4.78**	**5.98**	-0.14
	2	**-6.09**	**-5.91**	**2.13**	**5.02**
	3	-1.90	-1.54	**-2.17**	**4.06**
	4	-0.58	-0.57	-1.79	**2.24**
Num flake scars					
	0	**6.45**	**4.71**	**-10.30**	**4.73**
	1	0.81	**2.69**	**6.77**	**-8.62**
	2	-0.81	-0.12	1.62	-1.25
	3	-1.99	**-2.78**	-1.01	**3.19**
	4	-1.73	**-2.81**	-1.28	**3.58**
	5	-1.73	-1.35	0.81	0.75
	6	-1.54	0.10	-1.48	**2.18**
	7	-0.73	-0.73	1.50	-0.82
	8	-0.59	-0.57	1.09	-0.53
	9	1.57	-0.52	1.00	-1.55
Scar directions					
	0	0.96	-1.01	**-2.47**	**2.38**
	1	1.82	**2.78**	-1.78	-0.77
	2	**-2.72**	-1.85	1.46	1.35
	3	**-2.54**	-1.44	**6.12**	**-5.38**
	4	-1.05	-0.85	**3.08**	**-2.33**
Dorsal cortex					
	0	0.23	**-3.53**	**-6.21**	**7.28**
	25%	**-3.25**	-0.05	0.76	0.84
	75%	-0.58	0.94	**10.06**	**-9.66**
	100%	**5.96**	**3.29**	**-7.54**	**2.94**

SOM Table S19: Results of a Chi Square Test of a comparison of attributes on flaked pieces between Fazenda Matos, Lobi Bay and Plio-Pleistocene assemblages (significant results are highlighted in bold).

**Attribute**	**X2**	**df**	**p**
Exploitation direction	76.142	12	**<0.001**
Exploitation pattern	153.63	52	**<0.001**
Number of flaking surfaces	47.292	12	**<0.001**

SOM Table S20: Adjusted residual values for the Chi Square Test comparison of technological attributes on flaked pieces between Fazenda Matos, Lobi Bay and Plio-Pleistocene assemblages (significant results are highlighted in bold).

				Adjusted residuals		
**Attribute**		Fazenda Matos	Lobi Bay	Lomekwi	Oldowan >2Ma	Oldowan <2Ma
Exploitation direction	Unifacial	1.89	**2.09**	1.67	**-4.54**	**3.32**
	Bifacial	-1.23	**-2.51**	-1.27	**8.91**	**-4.66**
	Polyhedral	-0.58	-1.22	-0.42	**3.30**	**-2.14**
	Multifacial	-0.80	0.42	-1.28	**-2.12**	**2.25**
Exploitation pattern	USP	1.13	1.49	1.16	**-3.07**	1.45
	UAU1	0.32	1.71	0.49	-0.83	-0.47
	UAU2	**2.84**	1.17	-0.73	-1.18	-0.61
	UAUT	-0.86	-1.26	-0.51	**-2.83**	**4.03**
	UABI	-0.79	0.85	**4.02**	**-2.74**	1.43
	UP	-0.24	-0.38	-0.14	1.27	-0.92
	UC	-0.63	-0.85	-0.35	-1.22	**2.08**
	BSP	1.06	-1.55	-0.66	**3.20**	**-2.42**
	BAP	-1.38	-1.45	-0.82	**5.12**	**-2.85**
	BALP	-0.33	1.82	-0.22	0.32	-1.27
	BALT	-0.67	-0.93	-0.39	**-2.28**	**3.21**
	BP	-0.95	-1.49	-0.59	**5.16**	**-3.75**
	Polyhedral	-0.61	-0.88	-0.36	**3.30**	**-2.14**
	Multifacial	-0.75	0.42	-0.99	-1.77	**2.09**
Number of flaked surfaces	1	1.32	**2.33**	1.63	**-3.79**	1.80
	2	-0.55	-1.63	-0.94	**5.67**	**-4.10**
	3	-0.47	-0.76	-0.36	**-2.19**	**2.34**
	>3	-0.83	0.45	-1.22	-1.88	**2.42**

Supplementary Videos

SOM Video S1: Examples of yellow breasted capuchins (*Sapajus xanthosternos)* nutcracking at Fazenda Matos.

SOM Video S2: 3D video of refit set 1.

SOM Video S3: 3D video of refit set 2.

SOM Video S4: 3D video of refit set 3.

References

1. T. Proffitt, J. S. Reeves, D. R. Braun, S. Malaivijitnond, L. V. Luncz, Wild macaques challenge the origin of intentional tool production. *Science Advances* **9**, eade8159 (2023).

2. T. Proffitt, M. Haslam, J. F. Mercader, C. Boesch, L. V. Luncz, Revisiting Panda 100, the first archaeological chimpanzee nut-cracking site. *Journal of Human Evolution* **124**, 117–139 (2018).

3. T. Proffitt, *et al.*, Wild monkeys flake stone tools. *Nature* **539**, 85–88 (2016).

4. G. L. Isaac, The Archaeology of Human origins: Studies of the lower Pleistocene in East Africa. *Advances in World Archaeology, Academic Press* 1–87 (1984).

5. I. de la Torre, R. Mora, *Technological strategies in the lower Pleistocene at Olduvai Beds I & II* (Etudes et Recherches Archeologiques de l'Universite de Liege, 2005).

6. I. de la Torre, R. Mora, M. Domínguez-Rodrigo, L. de Luque, L. Alcalá, The Oldowan industry of Peninj and its bearing on the reconstruction of the technological skills of LowerPleistocene hominids. *Journal of Human Evolution* **44**, 203–224 (2003).

7. W. Andrefsky, *Lithic technology* (2008).

8. M. L. Inizan, M. Reduron-Ballinger, H. Roche, J. Tixier, *Technology and terminology of knapped stone* (C.R.E.P, 1999).

9. R. Mora, J. M. Moreno, X. T. Batlle, Un proyecto de análisis: el Sistema Lógico Analítico (SLA). *Treballs d'Arqueologia* 173–199 (1992).

10. T. Proffitt, Is there a Developed Oldowan A at Olduvai Gorge? A diachronic analysis of the Oldowan in Bed I and Lower-Middle Bed II at Olduvai Gorge, Tanzania. *Journal of Human Evolution* **120**, 92–113 (2018).

11. N. Toth, The oldowan reassessed: A close look at early stone artifacts. *Journal of Archaeological Science* **12**, 101–120 (1985).

12. Ž. Režek, H. L. Dibble, S. P. McPherron, D. R. Braun, S. C. Lin, Two million years of flaking stone and the evolutionary efficiency of stone tool technology. *Nat Ecol Evol* **2**, 628–633 (2018).

13. E. M. Finestone, *et al.*, New Oldowan locality Sare-Abururu (ca. 1.7 Ma) provides evidence of diverse hominin behaviors on the Homa Peninsula, Kenya. *Journal of Human Evolution* **190**, 103498 (2024).
